# BOLD fMRI activations and LC contrast associated with successful episodic memory in healthy older adults and MCI

**DOI:** 10.1002/alz.71682

**Published:** 2026-08-05

**Authors:** Lucia Penalba‐Sánchez, Yeo‐Jin Yi, Elif Kurt, Grazia Daniela Femminella, Clare Loane, Millie Duckett, Alina Zhunussova, Nikolaus Weiskopf, Martina F Callaghan, Ray Dolan, Robert J Howard, Emrah Düzel, Dorothea Hämmerer

**Affiliations:** ^1^ Institute of Cognitive Neurology and Dementia Research (IKND) Otto‐von‐Guericke University Magdeburg Magdeburg Germany; ^2^ German Center for Neurodegenerative Diseases (DZNE) Otto‐von‐Guericke University Magdeburg Magdeburg Germany; ^3^ Department of Neuroscience Aziz Sancar Institute of Experimental Medicine Istanbul University Istanbul Turkey; ^4^ Department of Translational Medical Sciences University of Naples Federico II Naples Italy; ^5^ Institute of Cognitive Neuroscience University College London (UCL) United Kingdom. London UK; ^6^ Department of Psychology University of Innsbruck Innsbruck Austria; ^7^ Department of Imaging Neuroscience UCL Queen Square Institute of Neurology University College London London (UCL) UK; ^8^ Max Planck Institute for Human Cognitive and Brain Sciences Leipzig Germany; ^9^ Felix Bloch Institute for Solid State Physics, Faculty of Physics and Earth System Sciences Leipzig University Leipzig Germany; ^10^ Institute of Psychiatry and the Wolfson Centre for Age Related Disease King's College London London UK; ^11^ Center for Behavioral Brain Sciences (CBBS) Magdeburg Germany

**Keywords:** brainstem, emotional processing, healthy aging, locus coeruleus (LC), memory encoding, midbrain, mild cognitive impairment, recognition memory, task functional magnetic resonance imaging

## Abstract

**INTRODUCTION:**

We examined whether locus coeruleus (LC) contrast, an indirect proxy for neuronal density and structural health, was related to delayed episodic memory in healthy older adults (OAs) and those with mild cognitive impairment (MCI) and its association with encoding‐related brain activity, controlling for emotional salience.

**METHODS:**

Participants (27 younger adults, 26 OAs, and 24 MCI) memorized emotional and neutral images during functional magnetic resonance imaging (MRI). Delayed memory was tested after 4 h and compared to immediate memory. LC contrast was measured via LC‐sensitive MRI.

**RESULTS:**

Memory was lower, independent of delay or emotional valence, and LC contrast was more strongly reduced in those with MCI compared to OAs. In OAs, LC contrast correlated with delayed memory and was mildly associated with LC activity during successful encoding. All groups remembered emotional images better than neutral ones, though memory was greatly impaired in those with MCI.

**DISCUSSION:**

LC contrast was associated with lower brain activity during encoding and emotional salience processing. Its relationship to delayed memory was evident in those with MCI.

## Highlights

- Emotional memory enhancement is largely preserved across adulthood and into early stages of cognitive impairment.
- OAs show greater engagement of the LC and basal ganglia during successful memory encoding.
- Individuals with MCI show no significant encoding‐related activation related to LC integrity and possibly reflecting limited compensatory capacity.
- A trend suggests higher LC activation in healthy seniors is linked to successful encoding, implying its role in supporting memory processes.
- This study highlights the underexplored role of the LC in age‐related and disease‐related memory processes.

## 1 BACKGROUND

Alzheimer's disease (AD) is characterized by progressive memory loss, typically linked to hippocampal dysfunction during episodic memory processes^1^, ^2^ and often preceded by neuropsychiatric symptoms and emotional dysregulation.^3^

Similarly affected early on in AD is the locus coeruleus (LC), the primary source of cortical norepinephrine neuromodulation.^4^ Its proximity to the fourth ventricle and exposure to small capillaries likely make the LC vulnerable to toxins^5^ and accumulation of tau pathology.^6^, ^7^, ^8^ Based on animal^9^ and human studies,^10^ tau pathology in the LC spreads progressively to the entorhinal cortex and neocortical regions via axons. This topographical progression of tau is a central mechanism in AD, influencing multiple cognitive domains.^6^, ^10^, ^11^, ^12^

LC neuronal loss in AD ranges from 30% to 80%, with reduced structural integrity evident even in MCI.^13^, ^14^, ^15^ This noradrenergic system deterioration is well established^16^ and highly implicated in cognitive decline.^8^, ^17^, ^18^

As the LC, through its widespread efferent projections to the hippocampus, helps modulate memory encoding of, in particular, emotional events, it is assumed to contribute to memory deficits in AD.^19^, ^20^ LC activation during aversive stimuli facilitates emotional memory encoding via projections to the amygdala and hippocampus.^21^, ^22^ Accordingly, lower LC integrity correlates with poorer episodic memory, particularly for negative stimuli, in healthy aging.^23^, ^24^

Despite these findings, the role of the LC in older adults (OAs) and AD during emotional processing remains unclear. Functional magnetic resonance imaging (fMRI) studies have reported increased amygdala activation in OAs in contrast to younger adults (YAs) when presented with positive emotional stimuli^25^ and reduced amygdala along with increased frontal activation with negative stimuli.^26^

Additionally, advances in ultra‐high‐resolution fMRI demonstrated dynamic LC activation during arousal‐related encoding, with LC responses following amygdala engagement before transitioning to hippocampal involvement during consolidation.^27^ LC activation also enhances hippocampal pattern separation at event boundaries, modulating how a continuous experience is segmented into discrete memories.^28^ Interestingly, OAs show increased LC activation during emotional salience processing and successful encoding compared to YAs, suggesting compensatory overactivation within a structurally compromised noradrenergic (NA) system.^29^ However, no study has yet compared LC functional activation directly during emotional processing and memory encoding across YAs, OAs, and individuals with MCI. Whether an impaired LC‐NA system in aging, and particularly in amnestic MCI, is the cause of reduced processing of negative stimuli requires further investigation.

In this study, we present a fMRI paradigm designed to activate the LC, similarly to the approach described elsewhere.^14^, ^29^, ^30^ By using a perceptual cover task, we assessed emotional salience processing with minimal demands on higher cognitive effort, making it particularly suitable for MCI. In addition, we combine task fMRI with LC‐sensitive structural MRI to examine how LC contrast ratio, which represents an indirect, MRI‐based proxy of neuronal density and structural health, relates to brain activation during episodic memory encoding and consolidation.^31^

We assessed the (1) LC contrast ratio, (2) immediate and delayed episodic memory for emotional and neutral scenes, and (3) related task‐based fMRI activation patterns, focusing on the brainstem and midbrain with an episodic memory task in YAs, OAs, and individuals with MCI.

Specifically, we expected, first, reduced episodic memory for neutral and emotional stimuli and reduced benefit from emotional valence in individuals with MCI compared to OAs and YAs likely to be related to lower LC contrast in individuals with MCI. Second, given a more intact LC‐NA system in YAs, we predicted stronger activations in regions implicated in negative affect, including the medial prefrontal cortex, anterior cingulate cortex (ACC), LC, amygdala, and insula.^33^ Additionally, we expected higher activation in mid‐temporal and frontal regions, hippocampus, and parahippocampus during successful encoding across all groups, with YAs showing stronger activations compared to OAs and MCI. Finally, we hypothesized increased LC activation in OAs compared to those with MCI during successful encoding, given a more intact LC structure in OAs.

**RESEARCH IN CONTEXT**

1. **Systematic review**: A PubMed review confirmed that memory‐encoding deficits in amnestic MCI are linked to functional changes in the hippocampus, adjacent regions, and frontal cortex. The contribution of the LC‐noradrenergic system remains underexplored. Emotional processing seems to decline with age and MCI; while psychological theories (e.g., positivity bias) are well studied, neurobiological hypotheses need further investigation.
2. **Interpretation**: Emotional enhancement of memory is broadly preserved across adulthood, though stronger in young adults alongside greater amygdala activation. Successful encoding engages the parahippocampus and precuneus in young adults, whereas older adults recruit the caudate, putamen, and LC. MCI patients show limited encoding‐related activation. Reduced LC integrity in MCI and higher LC activation in older adults for remembered items suggest LC integrity supports encoding.
3. **Future directions**: Future research should test noradrenergic interventions targeting the LC and combine imaging with biomarkers (CSF tau, amyloid, inflammation, vascular risk) to guide personalized treatment.

## 2 METHODS

### 2.1 Participants

A total of 93 individuals were initially recruited for the study. Data from 16 participants were excluded: two OAs and nine individuals with MCI who did not complete the delayed memory task, four MCI participants whose fMRI data were excluded due to task non‐compliance or inability to perform the task in the scanner (e.g., absence of button press responses or premature scan termination), and one MCI participant whose fMRI data were excluded due to excessive movement artifacts as assessed by a radiographer during the corresponding data collection session. The final sample consisted of 77 participants: 27 YAs, 26 OAs, and 24 OAs with MCI. Chi‐squared tests revealed no significant differences in gender distribution across groups (Table 1).

**TABLE 1:** Participant demographic, neurological, and cognitive descriptive information.

Group	YAs	OAs	MCI	ANOVA *p*
No., *n* (males)	27 (17)	26 (19)	24 (10)	χ^2^ ns
Age (SD)	23.1 (2.7)	70.6 (6.39)	72.6 (7.05)	<0.001(*f* 687)^a^ ^,^ ^b^
MMSE (SD)	29.52 (1.09)	29.48 (0.69)	25.49 (2.68)	<0.001 (*f* 44.06)^b^, ^c^
GMV (cm^3^) (SD)	1,180,181.37 (197059)	1,117,230 (200685.47)	1,064,780.88 (149937.10)	0.06 (*f* 2.77)^b^
LC int (SD)	0.13 (0.03)	0.16 (0.04)	0.10 (0.03)	<0.001(14.38)^d^

*Note*. Columns 2, 3, and 4 display results as mean (standard deviation). Column 5 shows chi‐squared for age and ANOVA *p* values.

Abbreviations: *n* = sample size; MMSE, Mental State Examination; GMV, gray matter volume; LC int, locus coeruleus integrity of the three voxels with maximum integrity (for LC int score see Methods); YAs, younger adult; OAs, older adult; MCI, mild cognitive impairment).

^a^ Significant differences between YAs and OAs.

^b^ Significant differences between YAs and those with MCI.

^c^ Significant differences between OAs and those with MCI.

^d^ Significant differences between all three groups.

Exclusion criteria included any self‐reported psychiatric or neurological disorders. All participants with MCI had received a formal diagnosis from a clinician and had a Mini‐Mental State Examination (MMSE)^32^ score between 22 and 27 (Table 1).

YAs were recruited via email from a participant database maintained by the Institute of Cognitive Neuroscience at University College London (UCL). OAs were recruited through advertisements, flyers, or clinical teams when attending memory clinics alongside friends or relatives with MCI. MCI patients were recruited through the Join Dementia Research database and from local memory clinics within the Camden and Islington National Health Service (NHS) Foundation Trust and the North East London NHS Foundation Trust.

Written informed consent was obtained from all participants. The study was conducted in accordance with the Declaration of Helsinki and received ethical approval from the UCL Research Ethics Committee (ethics reference number: 17/0091).

### 2.2 Study materials and paradigm

**FIGURE 1:**
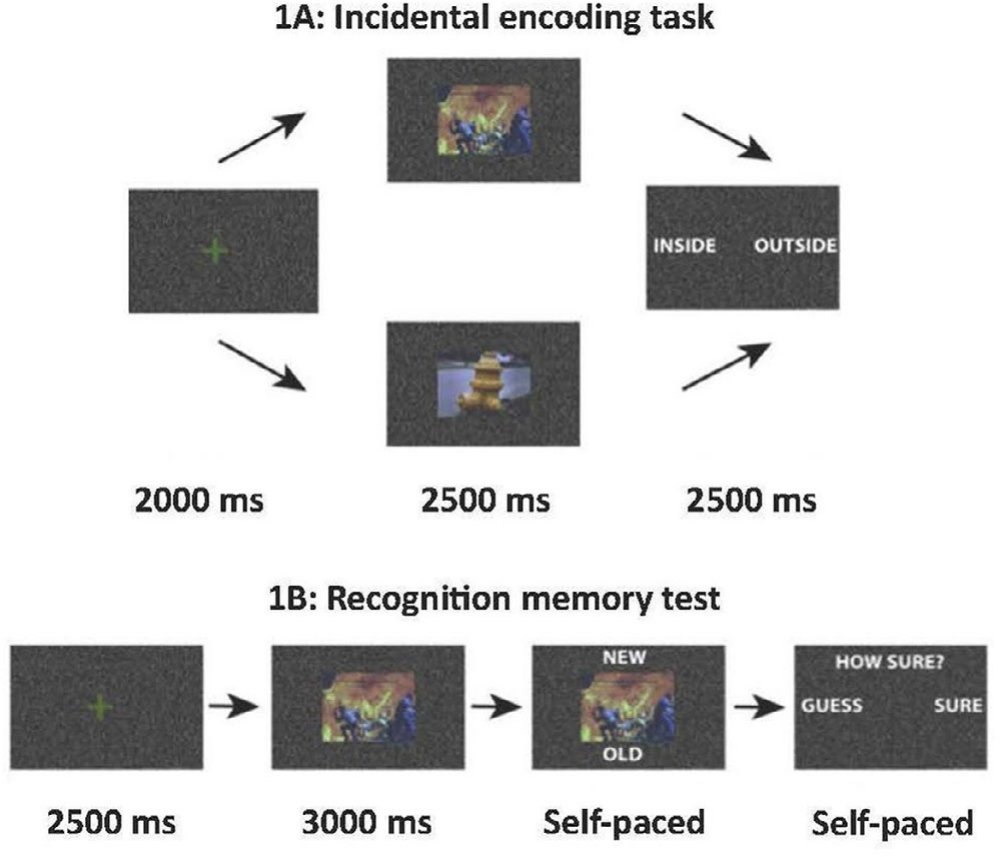
(A) Conducted inside scanner: Participants view an image of a scene, which may contain either negative or neutral affective content. As a cover task to ensure attentional processing of the scene, participants are instructed to press the right mouse button if the scene takes place outdoors (e.g., in a park or on a street) and the left mouse button if it takes place indoors (e.g., at home or in a restaurant). (B) Conducted outside scanner 30 min after encoding (immediate memory) as well as 4 h later (delayed memory) (*n* = 44 images previously shown in scan and *n* = 44 new images). Participants view an image and indicate whether it was previously shown during the scanning session (“old”) or if it is a novel image (“new”). Additionally, they specify their confidence level by indicating whether they “guess” or are "sure” as well as evaluate the emotionality of the scene as “very emotional” or “not very emotional.” Half of the trials displayed are emotional, half are neutral (*n* = 22 new emotional, *n* = 22 new neutral).

The task was programmed using MATLAB R2015a (version 8.5.0.197613, The MathWorks, Inc., 2015) and Cogent 2000 software. Scene images with both emotional and neutral content were drawn from a validated set taken from the International Affective Picture System (IAPS) database.^33^ Each scene image belonged to one of four groups, each containing 22 stimuli: emotional outdoor, emotional indoor, neutral outdoor, or neutral indoor. All stimuli were adjusted to 50% luminance to control for brightness effects on pupil dilation, which was recorded during the experiment; these results are not reported in this manuscript. Binary checkerboard noise patterns at 50% luminance served as background stimuli. Event‐related fMRI was recorded for the incidental encoding memory task (Figure 1A), followed by an immediate recognition memory test 30 min after encoding and a delayed recognition memory test 4 h after encoding (Figure 1B). Participants were asked to classify stimuli as indoor or outdoor to ensure sufficient attentional processing during the incidental encoding task. To ensure sufficient trial numbers for the analysis of subsequent memory effects,^34^ and given the expected memory decline and attentional strain in the MCI group, we combined trials correctly identified as “old” in either the immediate or delayed recognition test into a single “remembered” condition.

During the incidental encoding task in the scanner, 88 trials were presented: 44 emotional and 44 neutral. Scenes were presented for 2.5 s, preceded by a fixation cross which varied between 1.5 and 5.5 s (mean duration 2 s), followed by the outdoor‐indoor question, which was presented for 2.5 s. The total task duration was approximately 10 min. In the delayed memory test, 88 trials were presented: 22 new emotional images, 22 new neutral images, 22 emotional images previously shown during the scan session, and 22 neutral images previously shown during the scan session. Moreover, half of the scene stimuli were indoor and half outdoor. Scenes were presented for 3 s, preceded by a fixation cross which varied between 2.25 and 2.75 s (mean duration 2.5 s), followed by the old‐new question and guess‐sure question, which were presented until a response was given. Total task duration was approximately 12 min, depending on reaction times.

### 2.3 Imaging parameters

All MRI scans were acquired on a Siemens MAGNETOM Prisma (Siemens Healthcare, Erlangen, Germany) equipped with a 64‐channel head coil. Participants lay supine, with foam padding to minimize head movement. Sequences included both structural and functional acquisitions, as well as additional scans for geometric distortion correction.

#### 2.3.1 Structural MRI acquisition

Whole‐brain T1‐weighted structural images were acquired using a 3D Magnetization Prepared Rapid Gradient Echo (MPRAGE) sequence to support co‐registration of the functional images to structural group templates and evaluate individual volumetric indices (1.0 mm isotropic voxels, repetition time [TR] = 2530 ms, echo time [TE] = 3.34 ms, inversion time [TI] = 1100 ms, flip angle = 7°, field of view = 256 × 256 × 176 mm^3^). A dual‐echo gradient‐echo field map (TR = 1020 ms, TE1 = 10.00 ms, TE2 = 12.46 ms, flip angle = 90°, resolution = 3.0 × 3.0 × 2.0 mm) was acquired for B0 inhomogeneity corrections in functional recordings.

To assess the LC in the brainstem, a 3D magnetization transfer‐weighted (MTw) FLASH sequence (voxel size = 0.6 × 0.6 × 3.0 mm, TR = 24.50 ms, TEs = 3.35/6.15/8.95/11.75 ms, flip angle = 12°, field of view = 186 × 212 × 60 mm) was used to obtain high‐resolution images of LC‐sensitive, probably neuromelanin, contrast. The acquired MTw images were averaged across three repetitions and four echoes. It is assumed that neuromelanin deposits in the LC make the LC appear hyperintense in this so‐called “neuromelanin‐sensitive” contrast.^24^, ^35^ As neuromelanin accumulates within the LC cells, a higher signal intensity is interpreted as a higher cell density in a given voxel or higher structural integrity of the LC.^36^ As MRI measurements cannot completely ensure that the hyperintensity visible in this sequence is neuromelanin, we will refer to it as LC‐sensitive MRI. Quality assurance checks of acquisition were performed by the radiographer to prevent imaging data containing artifacts (e.g., movement artifacts). The sequence was repeated if an artifact was found during this visual inspection.

#### 2.3.2 Functional MRI acquisition

As the goal of the present study was to explore episodic memory and emotional processing with a focus on the brainstem and midbrain, data were optimized so that the resolution and coverage enabled the assessment of deep and small structures (e.g., LC and amygdala). A high‐resolution 2D T2*‐weighted whole‐brain multi‐band echo‐planar imaging (EPI) sequence was acquired (2.0 mm isotropic voxels, TR = 1450 ms, TE = 35 ms, acquisition time [TA] = 14:40, flip angle = 70°, multiband factor = 4, no in‐plane parallel imaging, bandwidth = 2246 Hz/Px, field of view = 212 × 212 × 144 mm, 72 interleaved slices). Image slices were acquired in an oblique axial orientation, tilted approximately 6.6° toward the coronal plane, with anterior to posterior phase encoding direction. This recording orientation and the 144 mm superior–inferior coverage ensured sufficient coverage of the midbrain and brainstem, including the LC, while minimizing signal dropout from air cavity near the brainstem.

#### 2.3.3 fMRI preprocessing steps

fMRI data were preprocessed in combination of SPM12 (Wellcome Centre for Human Neuroimaging, UCL, 2012; http://www.fil.ion.ucl.ac.uk/spm12.html), Advanced Normalization Tools (ANTs; version 2.5.1 post39‐g2236a75; http://stnava.github.io/ANTs/; preprocessing steps involving this toolbox are detailed in subsection 2.3.4 of this manuscript), FSL (version 6.0.1; https://fsl.fmrib.ox.ac.uk/fsl/, Analysis Group, FMRIB, Oxford, UK), and FreeSurfer (version 7.1; http://surfer.nmr.mgh.harvard.edu/, Martinos Center for Biomedical Imaging, Charlestown, Massachusetts). DICOMs were converted to NIfTI, and slice timing was corrected using the multi‐band parameters from the JSON sidecar of the fMRI sequence. Realignment and unwarping were performed with SPM12 using voxel displacement maps calculated from the double‐echo B_0_ field maps, with mostly default settings except for a spatial separation of 2 mm, a 3‐mm full width at half‐maximum (FWHM) realignment kernel, and fourth‐order interpolation given the higher‐than‐usual spatial resolution in acquired data. This step generated a mean functional image, which was later used as a representative image of a fMRI time‐series dataset during co‐registration. The 4D fMRI scans were then smoothed with a 2‐mm FWHM Gaussian kernel using the “smooth” function of SPM12. Then, first‐level contrasts were estimated in each subject's native space without registration or normalization. In addition, T1‐weighted structural images of all participants were bias‐corrected in ANTs (N4BiasFieldCorrection) prior to template generation. Study (a template created from all subjects) as well as three group‐specific templates (created including only YAs, OAs, and MCI patients) were constructed using antsMultivariateTemplateConstruction2^37^ (template construction details available at: https://github.com/alex‐yi‐writes/LC‐SpatialTransformation2021).

#### 2.3.4 Precise spatial transformations of structural and functional LC imaging data

To ensure sufficiently precise spatial transformations of structural and functional imaging data across subjects to examine activations in the small brainstem structures such as the LC, the procedures detailed in Yi et al.^38^ were modified and performed to fit the study objectives and dataset properties. First, individual structural images were non‐linearly transformed to the study‐specific and group‐specific templates via the antsRegistrationSyN.sh function to facilitate the normalization of mean functional images and the statistical contrast maps onto the template images.^37^ Then, mean functional images were rigidly registered to the corresponding structural images, also via antsRegistrationSyN.sh, generating affine transformation matrices. Finally, concatenating the transformations (affine matrices and warp fields) acquired from each step, the statistical contrast maps in their native space were transformed directly into the study‐ and group‐specific space with only one interpolation step using the antsApplyTransforms function of ANTs with linear interpolation setting.^37^

For functional LC data, a quality assessment procedure for sufficiently precise spatial transformations proposed by Yi et al.^38^ was subsequently employed. Specifically, six landmarks were manually placed in brainstem regions on individual mean functional images in four group‐ and study‐specific template spaces, i.e., study, YAs, OAs, and MCI templates. A rater with extensive experience in LC dataset evaluation performed this landmark placement (more details on the procedure of the landmark selection and placement protocols are described in Yi et al.).^38^ As shown in Figure S1, landmark deviations in the LC area were on average 0.79 mm and did not exceed the typical half‐width of the LC (2 mm).^39^

The LC appears as hyperintense voxels in the lateral floor of the fourth ventricle on T1‐weighted images.^40^, ^41^ Individual LC masks were manually segmented on 3D magnetization transfer‐weighted FLASH images (details of the sequence is described in Section 2.3.1) using the Paintbrush mode of ITK‐SNAP by two expert raters (YY, DH). Each rater independently identified hyperintense voxels bilaterally or unilaterally on consecutive axial slices as well as coronal slices, constraining selections to fewer than four voxels in plane (given the 0.6 × 0.6 mm native resolution) and across the rostrocaudal extent consistent with known LC dimensions from *post mortem* studies.^39^, ^42^ Final masks comprised the conjunction of voxels labelled by both raters. Sørensen–Dice coefficients for inter‐rater consistency across the three groups were 0.70 (YAs), 0.65 (OAs), and 0.57 (MCI), respectively. LC volumes and LC integrities were not correlated within groups (YAs: *r* = 0.22, *p* = 0.25; OAs: *r* = 0.19, *p* = 0.31; MCI: *r* = 0.28, *p* = 0.12), a result that is not surprising considering that volume indicates the LC extension while integrity is assumed to reflect neuronal density within the LC, which can decline differentially.^16^ See Supplementary Explanation 1 and Table S1 for detailed segmentation protocol and discussion of inter‐rater reliability.

Finally, we calculated a contrast ratio per participant, using the mean signal of the three brightest voxels in LC and pons reference masks as a more robust LC integrity measure.^43^

#### 2.3.5 Masks of small structures for second‐level analysis (small volume corrections)

For small regions of interest (ROIs), small volume corrections were applied during statistical analyses.^44^, ^45^ For this, region‐specific masks were generated for the group and study templates for the following areas: LC, thalamus, hippocampus, and amygdala. The LC masks were derived by transforming a meta‐LC mask published by Dahl et al.^46^ into each template space. For the thalamus, hippocampus, and amygdala, the masks were extracted by transforming the whole‐brain parcellation maps obtained from each participant's structural T1w images, processed using FreeSurfer's “recon‐all” function (version 7.4.1^47^).

### 2.4 Behavioral analysis and LC contrast

Memory performance (percent hits minus percent false alarms) of the encoded scenes and LC contrast values were computed and compared among the YAs, OAs, and MCI groups using one‐way ANOVA (*p* threshold < 0.05), followed by Bonferroni post hoc tests for pairwise comparisons. Pearson's correlations were conducted to examine the relationship between LC contrast and delayed memory performance, both overall (collapsed across neutral and emotional images) and separately for neutral and emotional images, in the whole sample as well as within subgroups (YAs, OAs, and MCI).

All the behavioral analyses were performed using SPSS version 29.

### 2.5 Task fMRI analysis: general linear model

To take into account atrophy differences across YAs, OAs, and MCI patients groups (cf. Table 1), two approaches to spatial transformation involving advanced warping methods (SyN‐based algorithms)^37^ were employed: (1) transforming images to a study‐specific template based on averages of anatomies across all three groups and (2) transforming images to separate group‐specific templates, based on averages of anatomies within each group. If possible, normalizing all participants into a shared space is preferable as it allows voxel‐level contrasts examining also partially activated ROIs between groups.^48^, ^49^, ^50^, ^51^ However, if, for example, the MCI group showed substantially smaller or fewer activated regions in the study‐specific template space (compared with its own group‐specific template), this might indicate registration distortions due to atrophy differences across groups, which affect significances at the group level in a joint template, in particular in a more atrophied group. To evaluate this, prior to investigating emotional and memory‐related contrasts, a standard “sanity‐check” contrast was evaluated comparing the study‐specific and group‐specific templates to ensure consistency of activation patterns in between study‐ and group‐specific templates. After confirming that both templates produced comparable results in motor‐related brain regions, for example, supplementary motor cortex and precentral gyrus (see results section), study‐template‐based analyses were used for the main emotional and memory contrasts. Event‐related regressors and contrasts of interest are summarized in Table 2. Additional models explored emotional processing and memory encoding while controlling for potential confounds or covariates (age, gray matter volume [GMV], and LC contrast; Supplementary Explanation 2).

**TABLE 2:** Event‐related GLMs, regressors and contrasts.

Event‐related GLM	Regressors	Contrast of interest
1. Motor (sanity check)	Emotional, neutral, indoor, fixation cross, response, remembered	Response > fixation cross
2. Emotional processing	Emotional, neutral, indoor, fixation cross, response, remembered	Negative images > neutral images
3. Memory encoding	Emotional, fixation cross, response, remembered, not remembered	Remembered images > not remembered images

*Note*. Table 2 shows GLM = event‐related general linear model; regressors =  covariates regressed in model; and contrast of interest = target contrasts.

Functional activations within and between pairs of groups were examined using one‐sample and two‐sample *t*‐tests in SPM12, respectively. For the sanity check contrast, the binarized study and group template gray matter masks were used to inspect whole‐brain‐level activations, while the binarized study template gray matter mask was applied for the emotional > neutral and remembered > not remembered contrasts. This approach excluded non‐gray matter tissues (i.e., cerebrospinal fluid [CSF] or white matter) from the results via an inclusion gray matter mask based on segmentations of the study/group templates. A threshold of *p*_uncorrected_ < 0.001 at the voxel level and *p*_uncorrected_ < 0.005 cluster level was used.^52^

For the more exploratory assessments of significant clusters in cortical and subcortical areas, we employed a two‐step cluster‐extent thresholding procedure to identify robust activations while balancing sensitivity and specificity. Statistical maps were initially thresholded at *p*_uncorrected _< 0.001. Then, data‐driven cluster extent threshold was defined by identifying a voxelwise false discovery rate (FDR) correction at *p *< 0.005 on the same contrast.^52^ The size in voxels of the largest FDR‐surviving cluster was noted. This FDR‐derived cluster extent threshold (k) was then applied to the initially thresholded map. Only clusters whose spatial extent exceeded this value are reported as statistically significant. This method warrants the idea that the reported clusters are not only spatially coherent but also meet a mathematically objective criterion for significance derived from the FDR framework.

For confirmatory small‐volume‐corrected (SVC) analyses of the thalamus, amygdala, LC, and putamen, significance assessments were corrected for multiple comparisons using family‐wise error correction (FWE), a more conservative measure for assessing the ratio of falsely rejected tests to all tests performed, with a voxel threshold of *p* < 0.01 as these regions are prone to presenting a lower signal‐to‐noise ratio. Findings reported as a trend were significant, with a *p* value between 0.05 and 0.1.

## 3 RESULTS

### 3.1 Structural LC results: contrast

**FIGURE 2:**
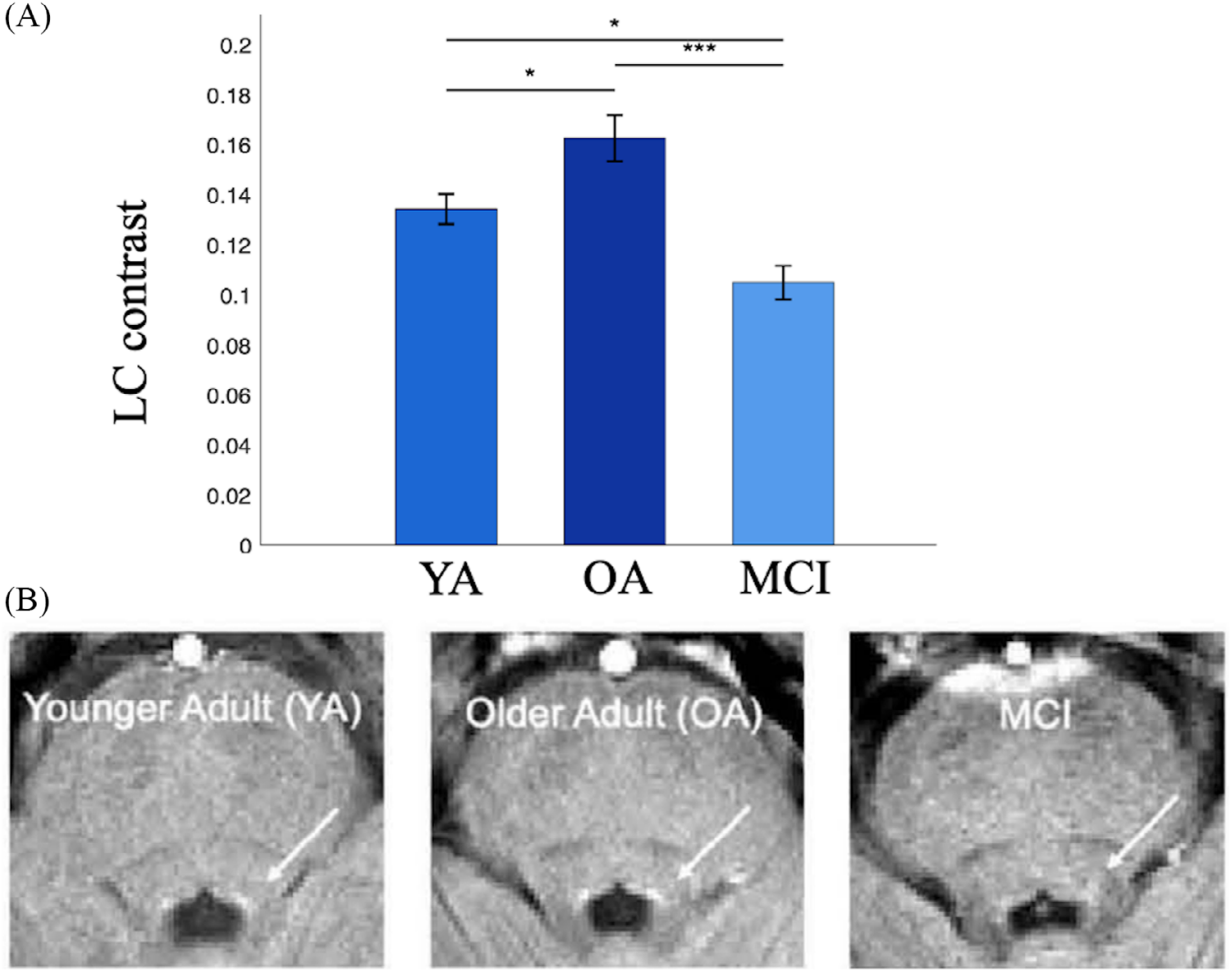
(A) Boxplots of statistical difference in LC contrast across groups. Lines with asterisks indicate statistically significant differences (younger adults < older adults; younger adults > MCI and older adults > MCI). (B) Example structural LC scans for one younger adult, one older adult, and one MCI patient, shown in mean across three repeated scans. The LC is shown as hyperintense area (white arrow).

As shown in Figure 2, LC contrast measured as the contrast ratio between the LC and a region of the pons differed between all three participant groups (*F*[2, 74] = 14.38, *p* < 0.001) and was larger in OAs compared to YAs (mean difference = 0.03, *p* = 0.02), larger in OAs compared to age‐matched individuals with MCI (mean difference = 0.06, *p* < 0.001), and larger in YAs compared to MCI (mean difference = 0.03, *p* = 0.02).

### 3.2 Behavioral results

**FIGURE 3:**
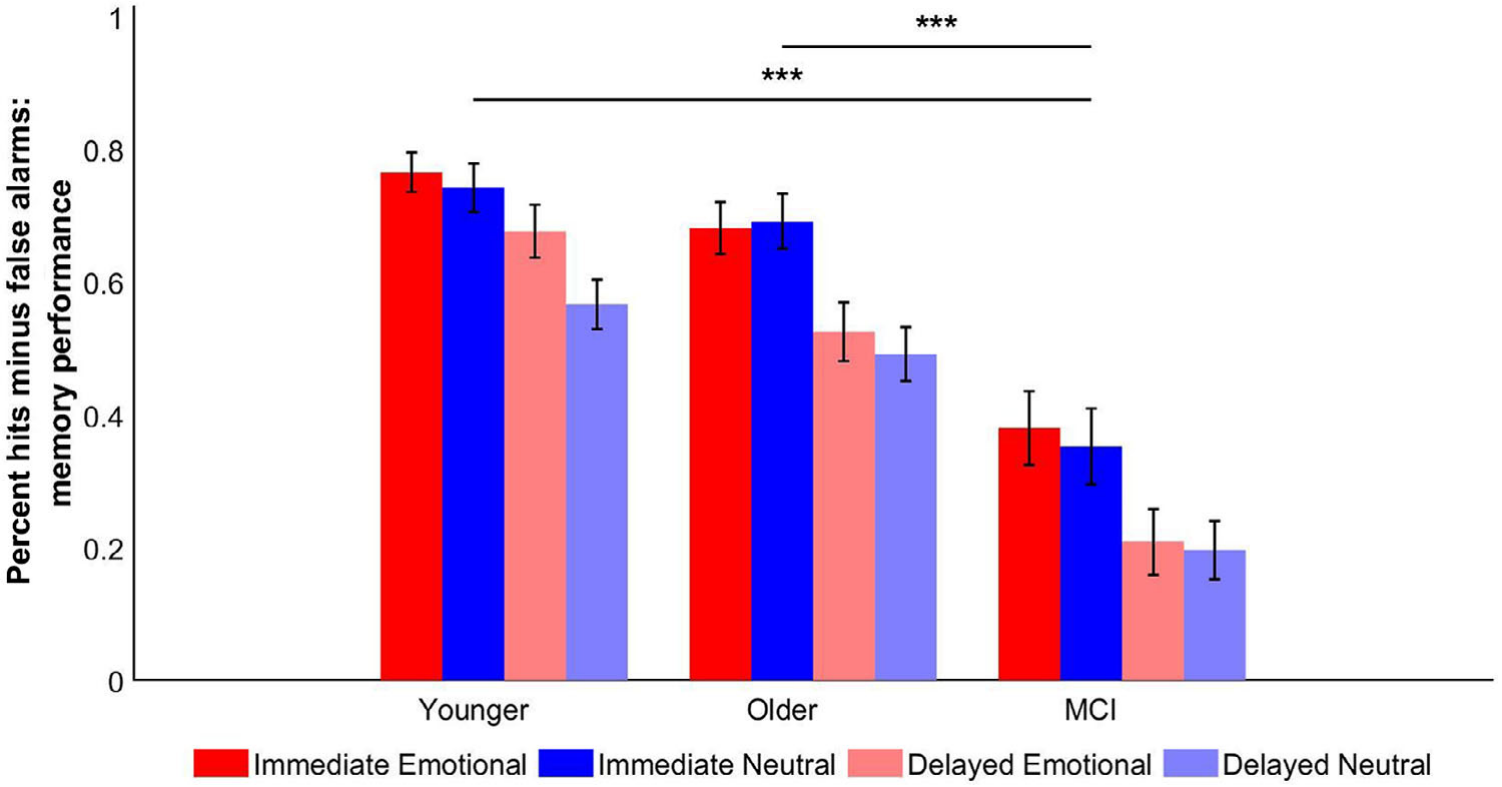
Mean memory performance (percent hits minus percent false alarms) of immediate (darker color) and delayed memory tests (lighter color) for each group and each type of image (negative emotional in red and neutral in blue). Lines with asterisks indicate main effect of group in memory performance (MCI < younger adults and older adults).

Behavioral results (Table S2 and Figure 3 in this manuscript) examine the recognition memory performance as operationalized by Hits – False Alarms considering the type of image memorized (neutral or emotional) and the time (immediate memory recognition and delayed memory recognition).

A 2 (type of image memorized: neutral, emotional) × 2 (time: immediate, delayed) × 3 (group: young, old, MCI) mixed‐effects ANOVA revealed a significant main effect of type of image memorized, *F*(1, 74) = 5.46, *p* = 0.022, indicating that emotional images were better remembered than neutral ones. There was also a significant main effect of time, *F*(1, 74) = 124.09, *p* < 0.001, suggesting that immediate recall was superior to delayed recall. However, the type of image memorized × time interaction was not significant, *F*(1, 74) = 1.93, *p* = 0.16. A main group effect was found, *F*(2, 74) = 30.66, *p* < 0.001, with post hoc Tukey tests indicating that MCI participants had significantly lower memory scores compared to both YAs (mean difference: 0.40, *p* < 0.001) and OAs (mean difference: 0.31, *p* < 0.001) groups, while YAs and OAs did not differ (mean difference: 0.09, *p* = 0.206). Interactions are reported in Table S2. Additional results on the relationship between pupil dilation during incidental encoding and consolidation are reported in Supplementary Results.

Motivated by reports that the LC supports long‐term memory formation,^4^, ^53^ we investigated the relationship between LC contrast and delayed recognition performance, collapsed across neutral and emotional images, as well as separately for neutral and emotional images. Pearson's correlations were calculated within each group, revealing a significant relationship for overall memory (i.e., neutral and emotional images collapsed) only in MCI patients (YAs: *n* = 25, *r* = 0.239, *p* = 0.25; OAs: *n* = 28, *r* = 0.110, *p* = 0.58; MCI: *n* = 24, *r* = 0.424, *p* = 0.04). However, pair‐wise comparisons of correlations using Fisher *z*‐tests revealed no significant group differences in the link between LC contrast and overall memory performance (YAs vs OAs, *z* = 0.46, *p* = 0.65; YAs vs MCI, *z* = 0.70, *p* = 0.48; OAs vs MCI, z = 1.15, *p* = 0.25). This suggests that the current sample sizes are too small to observe differences in links between LC contrast and delayed memory between groups. A similar pattern was found for neutral images: LC contrast correlated positively with delayed recognition in the whole sample (*n* = 77, *r* = 0.37, *p* < 0.001), with significance again only in the MCI group (MCI: *n* = 24, *r* = 0.41, *p* = 0.048; YAs: *n* = 27, *r* = 0.19, *p* = 0.34; OAs: *n* = 26, *r* = 0.06, *p* = 0.77). Fisher *z*‐tests indicated that the strength of the correlation did not differ significantly between groups (YAs vs OAs: *z* = 0.45, *p* = 0.65; YAs vs MCI: *z* = −0.80, *p* = 0.42; OAs vs MCI: *z* = −1.23, *p* = 0.22). Likewise, for emotional images there was a significant positive correlation in the full sample (*n* = 77, *r* = 0.389, *p* < 0.001), but within‐sample Pearson's correlation revealed a trend only in MCI (*n* = 24, *r* = 0.381,*p* = 0.06), with no significant correlation in YAs (n = 27, r = 0.252, p = 0.20), OAs (n = 26, r = 0.132, p = 0.52). Pairwise comparisons of correlations across groups using Fisher *z*‐tests revealed no significant differences: YAs versus OAs, *z* = 0.43, *p* = 0.67; YAs versus MCI, *z* = −0.48, *p* = 0.63; OAs versus MCI, *z* = −0.89, *p* = 0.37.

These results in emotional and neutral scenes indicate that although the correlations between LC contrast and memory consolidation are strongest in the MCI group, the strength of the associations does not differ significantly across groups (additional analysis in Figure S2). (For further results linking pupil dilation [PD] and delayed memory as well as PD and LC correlations see Figure S3 and S4.)

### 3.3 Task fMRI results

#### 3.3.1 Sanity check

Increased BOLD signal in motor areas were explored as a “sanity check” to assess whether activated areas in the three groups were affected by examining activations in a joint anatomical space across YAs, OAs, and MCI patients. They were assessed through a second‐level analysis of the contrast “response larger than fixation cross” to examine brain BOLD activations that are stronger when participants press any mouse button compared to when they passively view a baseline fixation cross. Note that these activations will also contain visual effects as responses were given during scene stimuli, which are more complex visually than fixation crosses.

On the study template, in the whole group (i.e., in a joint anatomical space across all three groups (YAs, OAs, MCI together), as expected, the contrast “response larger than fixation cross” revealed greater activation in motor regions such as the supplementary motor cortex, precentral gyrus, and parahippocampus, likely reflecting scene‐processing‐related effects. There were some differences across groups, with the precentral gyrus and supplementary motor cortex activated only in the YAs group, while the parahippocampus showed activation across all three groups.

Importantly, the activated ROIs of the “response larger than fixation cross” contrast remained largely consistent when examined in the group‐specific templates, except for the parahippocampus in the YAs group, which did not show a significant activation (Figure S5 and Table S3). This suggests that activations for the three participating groups can be examined and compared in the whole‐group anatomical space.

#### 3.3.2 Negative versus neutral images

The one‐sample *t*‐test results (study‐specific template; Table S4 and Figure S4A in this main manuscript) in the whole‐group analysis showed increased activation in regions associated with emotional processing, including caudate, amygdala left, mid/posterior cingulate cortex, the thalamus, and the inferior frontal cortex. Unexpectedly, the lingual gyrus and the primary visual cortex also showed heightened activation.

**FIGURE 4:**
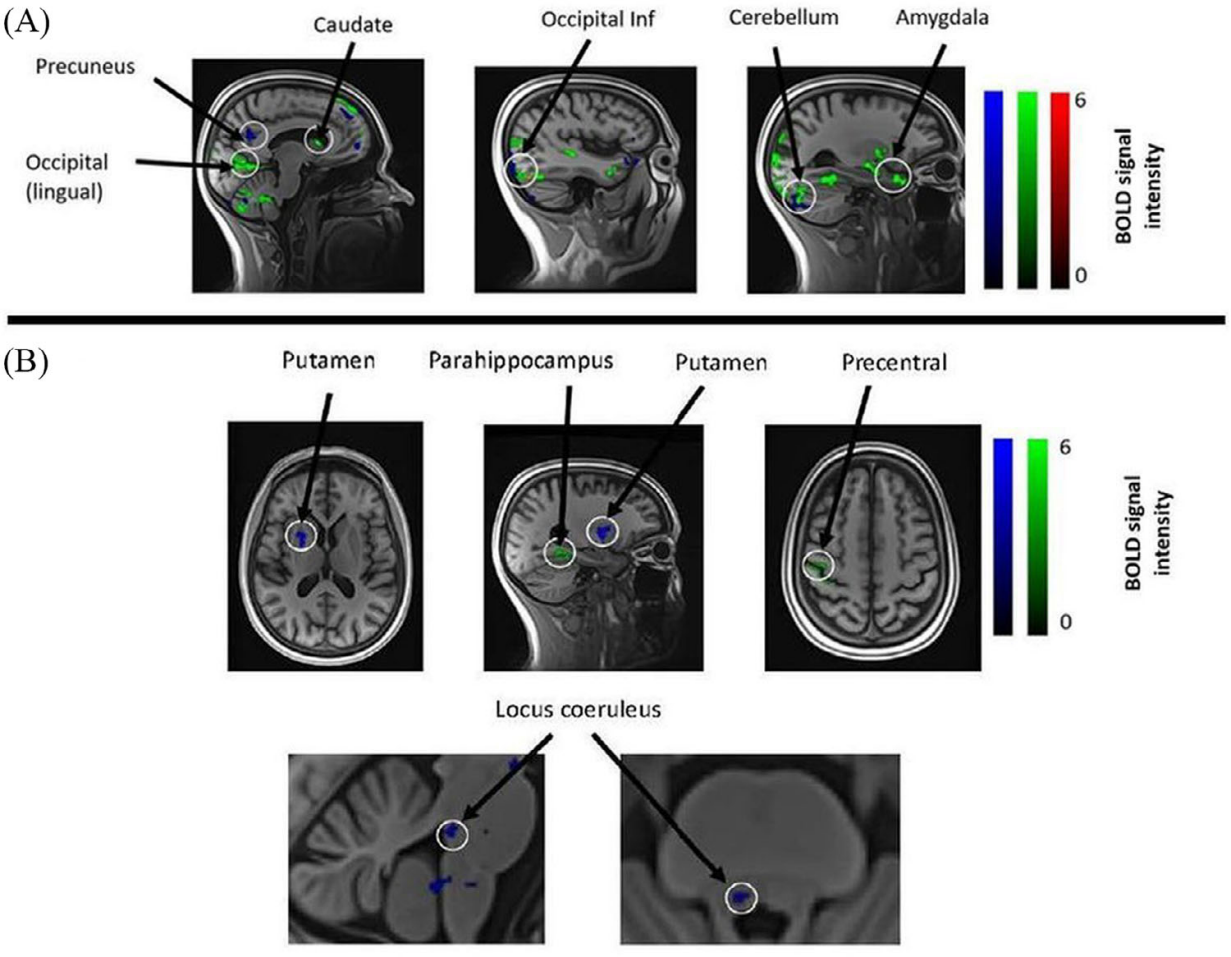
(A) Activations of contrast emotional > neutral scenes in study template. Activations of younger group (e.g., amygdala, cerebellum, caudate) in green of older adult group (e.g., occipital and temporal regions) in blue and of MCI patients (occipital inferior) in red. (B) Activations of contrast remembered > not remembered in study templates. Activations of younger group (e.g., parahippocampus) in green of older adult group (e.g., putamen) in blue. No significant activations were observed in MCI patients.

When assessing the groups separately, the YAs group showed increased activation in the insula, caudate, amygdala, hippocampus, and occipital inferior cortex. Occipital and temporal regions were activated in each group. Between‐group comparisons revealed no significant differences in activation between YAs versus OAs and OAs versus MCI (an overview of the results is displayed in Table 3 of this manuscript as well as in Table S4 and in Figure 4A in the main manuscript).

**TABLE 3:** Emotional processing larger than neutral.

Region	WG	YAs	OAs	MCI	YAs > OAs	OAs > MCI
Insula	No	−24, 25, −1	No	No	No	No
Caudate	−10, 28, 19	−20, 33, 20	No	No	No	No
Amygdala	−28, 12, −4	svc‐27, 11, −6	No	No	No	No
Praecuneus	No	−6, −44, 39	−10, −38, 22	No	No	No
Hippocampus	No	28, 11 ‐8	No	No	No	No
Lingual	−16, −36, −7	−13, −34, −7	No	No	No	No
Cingulate (mid‐posterior)	−8, −38, 25	1, −39, 35	No	No	No	No
Thalamus	svc: −20, −4, 13	No	No	No	No	No
Occipital inf	13, −54, −11	−36, −61, −30	−40, −65, −10	49, −42, −20	No	No
Frontal inferior	−44, 44, 5	−52, 46, 21	−47, 51, 16	No	No	No
Temporal	−44, −35, −26	−60, 2, 1	−56, −30, 14	−56, −42, 5	No	No
Cerebellum	No	11, −47, −45	−20, −43, −45	No	No	No

*Note*: Table 3 provides an overview of the brain regions that showed increased activation when participants processed emotional images compared to neutral ones. The study template was used. Only the significant results’ coordinates are reported. “No” indicates no significant activation. Results displayed reflect the one sample *t*‐test within‐group analysis (WG = whole group, YAs = younger adult, OAs = older adult, MCI = mild cognitive impairment) and the two‐sample *t*‐test or between‐group analysis (YAs > OAs, OAs > MCI). Occipital Inf = inferior occipital region. svc = result only significant when applying small volume correction. The statistical results are detailed in Table S3.

After controlling for the covariates age, GMV, and LC contrast, some results in the emotional > neutral contrast varied. In the YAs group, when controlling for age and GMV, the results were no longer significant. Conversely, most results remained intact after controlling for LC contrast, except in the lingual gyrus, insula, and fusiform. In the OAs group, no significant activations were observed when controlling for age and GMV. However, when controlling for LC contrast, all results remained stable except in the middle temporal, inferior temporal, and precuneus regions. Finally, the two regions that showed increased activation in the emotional contrast in MCI (i.e., inferior temporal and inferior occipital) did not show significant activations after controlling for any of the covariates.

Taken together, group‐wise analyses revealed that only the YAs showed stronger activations in the insula, caudate, cingulate, amygdala, and hippocampus for emotional compared to neutral stimuli, while all groups showed activations in the occipital inferior, suggesting enhanced visual processing of emotional stimuli and temporal regions. No significant between‐group differences were found when comparing YAs versus OAs, YAs versus MCI, or OAs versus MCI patients. These results suggest that while the networks engaged by emotionally salient stimuli are similar across age groups, the strength of activation does not differ significantly. Age and atrophy seem to explain the lack of specific effect for emotional processing (Table 3). Due to the reported link between pupil response to emotional/salient events and LC activations, additional analyses examining the association between pupil dilation and brain activations for the emotional larger than neutral contrast are reported in Supplementary Explanation 3. No relationship was observed in our sample.

#### 3.3.3 Remembered images larger than not remembered images

In the whole group, the contrast “remembered images larger than not remembered images” revealed increased activation only in the right putamen (*p*_FDRc‐ cluster corr) *p *= 0.05). When the groups were assessed separately, the YAs showed an increased activation in the parahippocampus right and left (*p*_FDRc‐ cluster corr) (*p* < 0.001), as well as in the precentral gyrus, cuneus, and precuneus. The OAs showed increased activation in the right and left putamen (*p* = 0.04 and *p *= 0.05, respectively) and a trend in the left LC small volume corrected (*x* = ‐6, *y* = ‐8, *z* = −27; *p*_FWEc = 0._08, *k*_E _= 4). No significant activations were observed in the MCI group. The lack of significant encoding‐related activation in those with MCI is consistent with their behavioral memory impairment and suggests a failure to engage the regions necessary for successful encoding.

When comparing results between pairs of groups, the precentral, cerebellum area 7b, and precuneus showed increased activation in the YAs in contrast to the OAs. The LC was slightly more active in the OAs compared to the MCI group (Figure S6.1). The cuneus, fusiform, and precentral showed increased activation in YAs > MCI in successful remembered > not successfully remembered (see Table S5).

There were no significant differences in the contrast “remembered larger than not remembered” in the following group comparisons: OAs larger than YAs, MCI larger than OAs, and MCI larger than YAs. An overview of the results is shown in Table 4 and Figure 4B of this main manuscript, as well as in Table S5.

**TABLE 4:** Remembered images larger than not remembered images.

Region	WG	YAs	OAs	MCI	YAs > OAs	OAs > MCI
Putamen	svc: 19, 29, 6	Svc:18, 25, 5	−23, 32, 8	No	No	No
Caudate	No	No	No	No	No	No
Parahippocampus	svc: 32, 1, −17	36, −13, −9	No	No	No	No
Precentral	No	35, −6, 41	No	No	35, −1, 54	No
Precuneus	No	No	No	No	11, −58, 34	No
Mid occipital	No	−21, −72, −5	No	No	No	No
Lingual	No	−29, −25, −16	No	No	No	No
LC	No	No	svc: −6, −8, −27*	No	No	svc: −6, −8, −27^*^

*Note*: Table 4 provides an overview of the brain regions that showed increased activation of the images displayed during the scan session that were subsequently remembered in the memory recognition test. The study template was used. Coordinates of the significant results are provided. “No” indicates no significant activation. Results displayed reflect the one‐sample *t*‐test within‐group analysis (WG = whole group, YAs = younger adult, OAs = older adult, MCI = mild cognitive impairment) and the two‐sample *t*‐test or between‐group analysis (YAs > OAs, OAs > MCI). Caudate: increased in OAs only after controlling for covariates. ^*^LC: locus coeruleus in OAs and OAs > MCI only marginal significant and not significant after controlling for covariates. Svc = results significant only when small volume corrected. The specific statistical results are detailed in Table S4.

Most of the results of the contrast “remembered larger than not remembered” remained significant after controlling for the covariates LC contrast, GMV, or age, with some exceptions: Within the OAs group, controlling for age and GMV revealed significant activation in the caudate (*p *= 0.04), which was not observed in the initial analysis. This suggests that accounting for interindividual differences in age and atrophy captures variance that occluded identifying larger activations in the caudate for remembered compared to not remembered stimuli, thereby revealing encoding‐related activity consistent with a compensatory role of the striatum in OAs.

Conversely, in the OAs and OAs > MCI comparison, the LC was no longer significantly activated in the remembered > not remembered contrast after controlling for age and LC contrast. An additional correlational analysis using SVC was performed to confirm whether LC contrast and LC activations in OAs were associated (Figure S6.2).

In conclusion, our results suggest that parahippocampus, precentral gyrus, and cuneus are implicated in successful memory encoding in the YAs group and differentiate memory encoding from that of the older group (OAs group and MCI), while the putamen, the caudate, and the LC are key in healthy aging.

## 4 DISCUSSION

The main objective of this study was to explore changes in OAs and those with MCI in episodic memory, associated brain functional activations, and the impact of LC contrast. Behavioral results showed comparable episodic memory performance between YAs and OAs, likely due to the easy memory task necessary to allow a comparative assessment with those with MCI. As expected, the MCI group showed poorer memory. Moreover, immediate recognition outperformed delayed, and emotional images were better remembered than neutral ones. However, no group difference based on emotional versus neutral memory effect was observed. With regard to group differences in LC contrast, we observed higher LC intensity in OAs compared to MCI. Moreover, LC signal was higher in OAs than YAs, consistent with the inverted U‐shaped trajectory of LC neuromelanin accumulation across the lifespan.^40^, ^54^, ^55^, ^56^, ^57^, ^58^ These intensity changes could also potentially point to unusually large differences in general cognitive reserve between YAs and OAs in our study (Supplementary Explanation 4). Changes in LC intensity over the lifespan have been associated with cognitive changes. While LC contrast is linked to working memory indirectly via the substantia nigra‐ventral tegmental area, and to fluid intelligence via the medial temporal lobe, it is directly associated with episodic memory.^23^ LC contrast was correlated with episodic memory across emotional and neutral scenes in the full sample, with stronger, albeit not significantly larger, links in the MCI group (Supplementary Explanation 5). Given our small sample size, it is possible that a specific emotional memory effect as well as exclusive links with LC contrast in MCI patients was occluded by heterogeneity in atrophy in other brain structures of the salience network supporting emotional memory. Moreover, recent studies suggest that a decline in LC integrity in AD might result in increased emotional reactivity, given difficulties in emotional regulation via a prefrontal‐brainstem loop.^59^ Interestingly, Zhunussova et al.,^60^ using the same dataset, observed generally increased pupil dilation in MCI patients compared to YAs and OAs, possibly corroborating the notion of a generally reduced arousal regulation (Figure S7). Future studies that include emotional ratings, graded emotional stimulation, and larger sample sizes of MCI patients will be required to investigate these speculations.

Our fMRI results provided insight into brain regions linked to emotional versus neutral processing. The amygdala, a region consistently linked with processing emotionally arousing stimuli,^61^, ^62^, ^63^ showed an increased activation only in the whole group and in the YAs group during the processing of negative emotional compared to neutral images. Despite the fact that there were no significantly larger activations in the amygdala in OAs or MCI patients, during emotional compared to neutral scenes, no significant age effects were observed in the preference for emotional over neutral images^64^ (Supplementary Explanation 6).

Apart from the relevance of the amygdala previously reported as a key region in emotional processing and memory encoding, further brain regions are assumed to contribute. In line with prior work,^65^, ^66^ the present study showed that the medial prefrontal cortex, inferior frontal cortex, inferior temporal cortex, striatal temporal cortex, lateral occipital cortex, and medial dorsal nucleus of the thalamus are activated in emotional scene processing. The insula, typically associated with emotional processing,^67^ showed an increased activation in YAs. We also observed enhanced activation in the lingual gyrus (YAs), precuneus, and primary visual cortex (all groups except MCI). Although not classic emotion regions, previous studies found higher visual cortex activation with emotional images, possibly reflecting greater attentional focus^65^, ^68^ (see Figure S8 for low‐level features assessment).

Several studies investigated the relationship between subjective arousal and objective physiological responses in OAs, revealing discrepancies. For instance, one study^69^ found that OAs reported higher subjective arousal in response to negative images compared to YAs, yet their electrodermal activity was significantly lower. Similarly, Mather et al.^70^ observed that OAs rated negative images as more arousing than did YAs, but their physiological responses did not increase correspondingly. This suggests a disconnect between subjective experience and objective arousal in OAs, highlighting the need to not only include physiological data such as pupil dilation and electrodermal activity but also self‐report data in future work.

In the contrast “remembered larger than not remembered,” the putamen was consistently activated in all three groups. As expected, the parahippocampus showed increased activation, albeit only in YAs; this effect was absent in OAs and MCI patients, likely due to greater atrophy and loss in function in older individuals.

Our results suggest, furthermore, that the precentral sulcus and the cuneus are involved in successful memory encoding in young participants. These findings align with previous studies that found that motor regions were activated during memory tasks that required a response action such as pressing a button.^71^ A meta‐analysis further identified that the precentral sulcus as part of the working memory network^72^ and both the precentral sulcus and cuneus were involved in visual and working memory.^72^, ^73^ A lack of activation in these regions has been shown in OAs and MCI groups, suggesting an aging‐related decline in their contribution. Conversely, the fusiform, which has been linked to the recognition of objects and faces,^66^, ^74^, ^75^ differentiates YAs from MCI patients, as the former group shows a higher activation.

Given the known role of the cuneus in visual processing and visual encoding,^72^, ^76^ its activation is likely related to the processing of scene stimuli. This process is crucial to forming connections with memory‐related regions like the hippocampus, the fusiform, and the amygdala, thereby strengthening memories. Reduced activation in these areas in OAs and MCI patients may contribute to their poorer memory performance. In addition to the cuneus, the precuneus, a region often associated with episodic memory, showed increased activation in successful memory encoding in YAs in contrast to OAs. In line with these results, a previous study indicated that highly vivid memories were associated with increased activation in the precuneus in Yas, in contrast to OAs.^77^ Additionally, a review of fMRI and positron emission tomography studies exploring the functional and behavioral role of the precuneus concluded that increased activation of this region was associated with higher memory performance.^78^ Interestingly, these findings were found not only in studies using a classic lab set of object stimuli, but also other types of naturalistic stimuli that enabled the assessment of episodic and autobiographic memory. This implies that the precuneus is activated when encoding any of these types of lab stimuli (scenes and objects) and that it is associated with episodic memory, which is impaired in early stages of amnestic MCI.

One of our main goals was to shed light on the functional role of the LC in memory encoding. The LC showed an increased activation (trend) in the OAs compared to MCI group and did not differ from the YAs group. A recent study showed that LC functional activation in OAs, in contrast to YAs, seemed to increase, especially when exposed to negative emotional images.^29^ A potential interpretation of these results is that healthy OAs recruit a partially compromised LC‐NA system to support memory function, a mechanism that may fail in MCI. Additional results linking PD are presented in Figure S4.

Finally, results that were significant or showed a trend did not remain significant after controlling for GMV and LC contrast, suggesting that interindividual differences in LC function are tied to interindividual differences in its structure. As shown in our behavioral and general linear model fMRI results, LC contrast and GMV appear to influence memory performance and LC activation during incidental encoding, regardless of diagnosis.

In conclusion, our study suggests that emotional salience enhanced memory across groups, despite an overall decline in MCI, indicating preserved emotional memory effects in pre‐clinical AD. Neural responses to negative stimuli shifted with age and pathology, from amygdala and insula engagement in YAs to greater temporal and occipital recruitment in OAs and MCI patients, consistent with compensatory or redistributed processing rather than loss of emotional responsiveness. Successful encoding showed reduced neural engagement in MCI despite preserved behavioral benefits, suggesting weakened memory‐related circuitry, atrophy, or reduced signal‐to‐noise ratio. LC contrast was reduced in MCI and predicted memory performance independently of emotionality, with the strongest structure–behavior relationship observed in MCI, supporting LC contrast as a sensitive marker of cognitive vulnerability in AD.

### 4.1 Limitations

This study has the following limitations. First, the modest sample size and MMSE heterogeneity in the MCI group limits generalizability, and null fMRI findings should therefore be interpreted cautiously. Second, the absence of biological markers (e.g., CSF, amyloid, genetic risk) limits insights about underlying pathology. Third, we did not assess subjective or physiological indices of emotional engagement or control for image memorability factors that may influence encoding beyond valence. Finally, the lack of significant emotional processing‐related activation in MCI despite preserved behavioral effects may reflect the limitations of standard fMRI contrasts in capturing LC–NA engagement or compensatory processes.

## CONFLICT OF INTEREST STATEMENT

All authors certify that they have no affiliations with or involvement in any organization or entity with any financial or non‐financial interest in the subject matter or materials discussed in this manuscript. Author disclosures are available in the Supporting Information.

## CONSENT STATEMENT

Informed consent was obtained from all subjects involved in this study.

## Supporting information



Supporting Information

Supporting Information
