# Supplementary material for: BOLD fMRI activations and LC contrast associated with successful episodic memory in healthy older adults and MCI
Source: Alzheimers Dement. 2026 Aug 5;22(8):e71682. doi: 10.1002/alz.71682 (PMC13439747; doi:10.1002/alz.71682)
Supplement: Supplementary file 2 [file ALZ-22-e71682-s002.docx]

**Supplementary**

**Table of contents**

- **Explanations:**
  - **Explanation 1. Methods: LC segmentation protocol and inter-rater reliability**
  - **Explanation 2. Methods: fMRI GLM second level analysis covariates**
  - **Explanation 3. Results: fMRI pupil dilation and brain activations contrast emotional > neutral**
  - **Explanation 4. Discussion: LC contrast changes over the lifespan**
  - **Explanation 5. Discussion: LC contrast, episodic memory consolidation and pupil dilation (PD)**
  - **Explanation 6. Discussion: Formation of emotional episodic memory**
- **Figures:**
- **Figure 1. Histograms landmarks**
- **Figure 2. Relationship between LC contrast ratio and delayed memory**
- **Figure 3. Pupil dilation during encoding and delayed memory performance**
- **Figure 4. Pupil dilation during encoding and LC contrast ratio**
- **Figure 5. Sanity check**
- **Figure 6. LC BOLD fMRI during incidental encoding in older adults (OA)**
- **Figure 7. Pupil dilation**
- **Figure 8. Low-Level Visual Features by condition**
- **Tables:**
- **Table 1. Inter-rater variability LC segmentation**
- **Table 2. Behavioural results: within-Subjects Effects and group interaction effects**
- **Table 3. Sanity check response larger than fixation cross (motor)**
- **Table 4. Significant activations of the contrast Emotional Processing (Negative vs. Neutral scenes)**
- **Table 5. Significant activations Remembered Images Larger than not Remembered Images (memory)**

**Explanations:**

**Explanation 1. Methods: LC segmentation protocol and inter-rater reliability**

LC segmentation was performed on 3D magnetisation transfer-weighted (MTw) FLASH images, which provide superior LC contrast ^[1]^compared to conventional T1-weighted sequences ^2^. Two expert raters (YY, DH) independently segmented each participant's LC using ITK-SNAP's manual segmentation tools, blind to group membership.

Anatomical criteria: The LC was identified as hyperintense voxels located in the lateral floor of the fourth ventricle, adjacent to the superior cerebellar peduncle, consistent with established anatomical landmarks from histochemical and post-mortem studies ^3–5^.

Spatial constraints: Given the native in-plane resolution of 0.6×0.6 mm in our MTw scans, raters selected no more than four contiguous voxels in the axial plane to remain within the known LC width of approximately 2.5 mm ^3^. Segmentation proceeded slice by slice across the rostrocaudal extent of the LC (approximately 12-17 mm; German et al., 1988)^6^.

Conjunction approach: Final LC masks were extracted from the conjunction (logical AND) of voxels labelled by both raters, ensuring conservative estimates that minimised false positives.

Inter-rater reliability: Sørensen–Dice coefficients indicated moderate to good inter-rater agreement (YA: 0.70; OA: 0.65; MCI: 0.57). Lower agreement in MCI likely reflects reduced LC contrast and increased anatomical variability due to neurodegeneration, which complicates LC segmentation. Comparable Dice values have been reported for brainstem structures in neurodegenerative populations (e.g., Parkinson’s disease: 0.49; Alzheimer’s disease: 0.51). Importantly, LC contrast is generally a more informative measure of interindividual differences in LC structural integrity than LC volume, particularly in healthy aging and prodromal stages of neurodegeneration. MRI LC signal intensity, assumed to scale with neuronal density, provides a sensitive integrity marker, whereas volumetric estimates are more strongly affected by partial volume effects at current MRI resolutions. Consistent with this, LC intensity has been linked to cognitive and structural brain measures in prior work, while LC volume shows less consistent associations. To mitigate the impact of differential inter-rater agreement, we used a conservative conjunction mask requiring voxel-wise agreement between raters, prioritizing specificity. LC volume and LC were uncorrelated within all groups (YA: r = .22; OA: r = .19; MCI: r = .28; all p > .12), indicating that LC contrast ratios were not driven by mask size. In addition, LC contrast was estimated using the three most intense LC voxels per individual to further reduce potential dilution effects. Finally, LC contrast ratios did not differ systematically between individuals with lower- versus higher-than-mean Dice coefficients within any group, for either the full conjunction mask or the max-three-voxel approach (See in this Supplementary Table 1), suggesting that group differences in Dice scores did not substantially bias LC contrast estimation.

**Explanation 2. Methods: fMRI GLM second level analysis covariates**

Including age, grey matter, and LC intensity as covariates enabled us to explore whether interindividual variability in functional activation in the LC is related to interindividual differences in age, atrophy, and anatomical LC variability.

**Explanation 3. Results: fMRI pupil dilation and brain activations contrast emotional > neutral**

Additional analyses were conducted to assess the relationship between pupil dilation (PD) in response to emotional images and blood oxygen level dependent signal (BOLD) activations in the insula, caudate, amygdala, and locus coeruleus (LC) across individuals, both in the whole sample and within each group. These analyses were performed using an SPM one-sample *t*-test on the emotional > neutral contrast, with pupil dilation following the presentation of emotional scenes entered as a covariate.

In the whole sample, no significant links were observed between pupil dilation and BOLD activations in any of the regions of interest. Similarly, within the YA group, pupil dilation was not associated with activation in the LC, amygdala, caudate, or insula.

**Explanation 4. Discussion: LC contrast changes over the lifespan**

Several studies have shown that LC signal intensity starts to increase from early adulthood, reaches apex around age 60, and gradually declines thereafter. Given that our YA group (age range 20-28) is positioned on the ascending half of the curve and our OA group (age range 60-82) is near or slightly past the apex point, higher LC signal in OA relative to YA is the expected pattern in healthy ageing

In addition to this expected lifespan trajectory, it is important to note that LC contrast estimates may be influenced by methodological factors inherent to LC sensitive MRI. In particular, contrast ratios depend on both LC signal intensity and signal characteristics of the pontine reference region, which may itself be affected by age-related changes in tissue composition or noise properties. However, in the present sample, raw pons signal intensity did not differ significantly between younger and older adults, and LC contrast ratios correlated with raw LC signal but not with pons signal intensity, suggesting that group differences in LC contrast were primarily driven by LC signal rather than reference-region effects.

Another possibility is that older adults in the present sample exhibited relatively preserved LC structure, consistent with prior reports linking higher LC contrast to better cognitive health in aging. Although we did not include detailed cognitive assessments that would allow us to directly test this interpretation, the observed pattern does not appear to be attributable to trivial normalization artefacts. Taken together, while the finding of higher LC contrast in OA compared to YA should be interpreted with caution, it is broadly consistent with known neuromelanin accumulation dynamics and does not appear to be driven by age-related confounds in the reference region.

**Explanation 5. Discussion: LC contrast correlated, episodic memory consolidation and pupil dilation (PD)**

Although in the within group analysis only the MCI group showed an association between LC contrast and episodic memory consolidation. These results require careful interpretation as additional analysis suggest that the relationship between LC contrast and memory consolidation remains significant after controlling for group. The small sample size could be limiting the statistical power. Notably, although emotional images were remembered better than neutral images across all groups, indicating preserved emotional memory enhancement, LC intensity in MCI was most strongly associated with delayed memory for neutral scenes. This suggests that a reduced LC contrast does not eliminate emotional memory enhancement. Emotional memory is supported by a distributed network involving amygdala–hippocampal interactions and broader salience-related circuits, and these pathways may remain relatively preserved or compensate when the LC declines. From a biological perspective, the preservation of emotional memory enhancement in early pathology by means of alternative regions/networks is plausible, as encoding emotionally salient or threatening information is essential for survival.

At the same time, LC contrast was associated with overall memory performance independent of stimulus valence, indicating that LC-related neuromodulatory processes support general mnemonic capacity rather than emotional memory selectively. Notably, this structure–behavior relationship was most evident in the MCI group, suggesting that residual LC intensity becomes behaviorally relevant when the system is compromised. In this context, LC integrity may act as a non-specific reserve mechanism that supports encoding and consolidation across stimulus types, particularly for neutral, less salient information that lacks strong emotional or motivational support from other neural systems. Thus, while emotional memory enhancement may be sustained by non-LC circuits in MCI, LC intensity appears to contribute to maintaining baseline mnemonic function in early pathology.

To complement these findings, we conducted additional analysis with PD data. Interestingly, increased PD during delayed recognition memory was associated with successful consolidation of emotional scenes and a trend for neutral scenes only in MCI (Supplementary Figure 3.3). This association, however, was not seen between PD during encoding and successful memory performance. These results, along with patients’ reports during testing confirmed that they were emotionally affected and might have had difficulties regulating their emotions.

**Explanation 6. Discussion: Formation of emotional episodic memories**

Forming emotional episodic memories is a dynamic phenomena, i.e., arousal mediated consolidation, that involves not only the MTL memory system but also the amygdala. The co-activation or lack of it of these regions over time (from processing the stimuli until recalling them minutes, hours or days after) enables the prediction of consolidation emotional experiences^7^. There is a lack of activation in the amygdala in the MCI group, however maybe connectivity in this case could predict their slightly better performance in encoding of emotional scenes. Future studies using higher fMRI field strength, analysis that capture delays in encoding e.g., dynamic activation and connectivity and larger samples may capture salience-related brain activations more reliably.

**Figures:**

**Figure 1.** Histograms of in-plane distances between landmarks defined on the study-specific template and single-subject landmarks delineated on mean functional images transformed into the template space. Each inset in the corresponding histogram plot indicates its anatomical position on the structural MNI template. The detailed procedure for selecting and placing the landmarks, as well as quantifying the distances, is described in Yi et al.'s (2023) work. Note that the distances in the Outline Brainstem landmarks vary, as they were placed anywhere along the outline of the brainstem border. The mean and standard deviation distances for landmarks are described underneath the anatomical position insets.

1.1. **Histograms landmarks (study template)**

1.2. **Histograms landmarks (younger adults’ template)**

1.3. **Histograms landmarks (older adults’ template)**

1.4. **Histograms landmarks (MCI template)**

**Figure 2. Relationship between LC contrast ratio and delayed memory**

2.1. A multiple linear regression was conducted to examine the impact of LC on delayed global memory performance (i.e., emotional and neutral scenes recognised collapsed) even after controlling for group (β = 1.16, p = 0.045). Group differences were also significant, with MCI group showing the largest deficit relative to the YA. The model explained 45–48% of the variance (R² = 0.475, adjusted R² = 0.454), indicating a moderate to strong fit. These results suggest that higher LC contrast is associated with better global memory independently of group differences.

2.2. A multiple linear regression model to explore the relationship between LC contrast ratio and emotional memory performance controlling for age showed a statistically significant result, F(3,73)=21,p<.001, accounting for 46.3% of the variance in emotional memory (R² = 0.463, adjusted R² = 0.441). LC contrast ratio significantly predicted emotional memory, β=1.31, SE = 0.64, t(73) = 2.04, p = .045, indicating that higher LC contrast was associated with better emotional memory. Group also had significant effects: compared to the YA, the OA showed lower performance (β=-0.165, SE = 0.062, t(73) = -2.67, p = .009), and MCI showed even lower performance (β=-0.418, SE = 0.064, t(73) = -6.50, p < .001). LC contrast positively influences emotional memory independently of group.

2.3. Regarding delayed memory performance of neutral scenes, the model was significant F(3,73)=15.6, p<.001, explaining 39.1% of the variance (R² = 0.391, adjusted R² = 0.366). LC contrast ratio did not significantly predict neutral memory, β=1.01, SE = 0.61, t(73) = 1.66, p = .101. Group had a significant effect only for the MCI group in contrast to the YA (β=-0.333, SE = 0.061, t(73) = -5.48, p < .001), while the OA did not differ significantly from the MCI (β=-0.092, SE = 0.059, t(73) = -1.57, p = .120).

**Figure 3. Pupil dilation during encoding and delayed memory performance**

3.1. No significant correlations were found between pupil dilation during emotional scenes in the incidental encoding task and hit rate minus false alarms for emotional scenes in the delayed recognition test in YA (r = 0.084, p = 0.717), OA (r = 0.203, p = 0.377), or MCI patients (r = 0.062, p = 0.785), as revealed by Pearson's correlation analyses.

Hits minus FA emotional scenes

3.2. Pearson's correlation analyses showed no significant correlations between pupil dilation during neutral scenes in the incidental encoding task and hit rate minus false alarms for neutral scenes in the delayed recognition test in YA (r = -0.019, p = 0.933), OA (r = 0.005, p = 0.982), or MCI patients (r = 0.066, p = 0.771).

Hits minus FA neutral scenes

3.3. Pearson's correlation analyses between successful delayed memory performace (hits minus false alarms) of emotional scenes and pupil dilation revealed no significant associations in YA (r = 0.121, p = 0.584) or OA (r = -0.118, p = 0.610). However, MCI patients showed a significant positive correlation (r = 0.449, p = 0.041), indicating that **better performance** was associated with greater pupil dilation responses for emotional scenes in the delayed recogntion test in this group.

3.4. Pearson's correlation analyses revealed no significant associations between **delayed memory performance (higher hit rate minus false alarms) and** pupil dilation responses for neutral scenes in the delayed recogntion test across any group: YA (r = 0.209, p = 0.326), OA (r = 0.149, p = 0.507), and MCI patients (r = 0.296, p = 0.192).

**Figure 4. Pupil dilation during encoding and LC contrast ratio**

4.1. Pearson’s correlation analyses revealed no significant associations between pupil dilation during emotional scenes in the incidental encoding task and LC contrast across any group: YA (r = 0.073, p = 0.749), OA (r = 0.294, p = 0.184), and MCI patients (r = 0.029, p = 0.898).

4.2. Pearson’s correlation analyses revealed no significant associations between pupil dilation during neutral scenes in the incidental encoding task and LC contrast across any group: YA (r = 0.35, p = 0.114), OA (r = 0.3, p = 0.158), and MCI patients (r = -0.04, p = 0.865).

**Figure 5. Sanity check**

Functional brain activations of the sanity check motor contrast (response > fix). Results are compared in study template across all three groups (top left), in study template space for individual group results (top right) as well as in group-specific templates (bottom). Activations in younger adults are indicated in green, in older adults in blue, and in MCI in red. Sanity checks examined differences in activated brain areas in group-specific and study template space which would suggest distortions due to merging across group-specific anatomies. Group-specific activation patterns were comparable in study and group-specific space, suggesting that between group comparisons in study space are not strongly affected by anatomical differences between groups (Supplementary table 1).

**Response > fixation cross** (study template)

Overlay young, older, MCI (study template)

Whole group

PHG

Precentral

6

0

6

1

Young, older, MCI (group template)

6

0

Discussion sanity checks:

As expected, motor-related brain regions exhibited enhanced event-related BOLD activation in the sanity check. For instance, the supplementary motor cortex and precentral gyrus were activated during finger-tapping tasks, particularly in the younger group. Interestingly, the parahippocampus was additionally significantly activated in all three groups, likely reflecting its role in scene processing. As the majority of areas identified in group-specific templates are also observed in the study template, it can be assumed that anatomical distortions in bringing individuals in the joint study template space is not affecting the interpretability of between group comparisons in study space substantially.

**Figure 6.** LC BOLD fMRI during incidental encoding in older adults (OA)

6.1. The figure below shows in blue the the BOLD fMRI activations in the left LC during incidental encoding (contrast remembered > not remembered) in older adults (OA) using small volume correction (SVC) (FEW-corrected *p* = 0.082, t = 2.46, df =24). The LC mask (study template) can be seen overlaid in red. Image displayed in neurological convention view, coronal, axial and sagittal views.

6.2. An additional correlational analysis using small volume correction (SVC) was performed to confirm whether LC contrast and LC activations in OA are associated. The results showed a small effect, with a trend of a positive link between structure and activation in this region (SVC, cluster-level FWE-corrected *p* = 0.083), t = 2.57, df =24). This suggests that interindividual differences LC contrast contribute to differences in LC function during incidental memory encoding.

**Figure 7. Pupil dilation**

The figure below, published by Zhunussova et al., (2025)^8^ shows changes in pupil dilation (PD) from 1 to 2 seconds after stimulus onset during an incidental memory task involving emotional and neutral scences (same participants and visit as the ones in the current study). Younger and older adults (YAs and OAs) showed greater mean pupil dilation in response to emotional compared with neutral stimulli. In contrast, participants with MCI exhibited increased PD in contrast to YAs and OAs, regardless of scene type. While speculative, it is possible that these increased PC in the context of emotional stimuli may reflect a general deficit in arousal regulation in MCI patients.

**Figure 8. Low-Level Visual Features by condition**

It is possible that visual features like (1) mean luminance, (2) root-mean-square (RMS) contrast, (3) and colour saturation might have differed between emotional and neutral images and therefore affected the observed activations in addition to assumed attentional effect.

To address this concern, we extracted and compared three key low-level features between emotional and neutral images, which are (1) mean luminance, (2) root-mean-square (RMS) contrast, (3) and colour saturation.

Independent samples *t*-tests revealed no significant differences between emotional (*N*=120) and neutral (*N*=120) images for any of these features. Mean luminance did not differ between conditions, *t*(238)=1.24, *p*=0.214, Cohen’s *d*=0.16), nor did RMS contrast, *t*(238)=1.15, *p*=0.251, Cohen’s *d*=0.15, or colour saturation, *t*(238)=-0.23, *p*=0.817, Cohen’s *d*=-0.03. All effect sizes were small (|*d*|<0.2), indicating that the emotional and neutral image sets were well matched on these basic visual properties. These results suggest that the observed differences in visual cortex activation for emotional relative to neutral images are unlikely to be driven by low-level visual feature confounds.

The codes that performed this analysis can be inspected via this link: <https://github.com/alex-yi-writes/public_codebase/blob/main/LiDO_EM_lowlevel_visfeatures_v2.m>

**Tables:**

| **Table 1. Inter-rater variability LC segmentation**  **Comparison of the mean LC CR low Dice score and LC CR high Dice score** | | | | | | |
| --- | --- | --- | --- | --- | --- | --- |
|  | Conj mask: LC CR low Dice | Conj mask: LC CR high Dice | Difference  high – low Dice | Conj mask, max3: LC CR low Dice | Conj mask, max3: LC CR high Dice | Difference high-low Dice |
| Younger adults | 0.0928 | 0.0893 | **-0.0035** | 0.1310 | 0.1319 | **0.0009** |
| Older adults | 0.1254 | 0.1083 | **-0,0171** | 0.1649 | 0.1581 | **-0,0068** |
| MCI patients | 0.0786 | 0.0806 | **0.0020** | 0.0974 | 0.1079 | **0,0105** |
| Note. The table shows the differences in Locus coeruleus (LC) contrast ratio (CR) between low and high Dice across groups for the whole conjunction mask as well as the max3 voxels in the conjunction mask. | | | | | | |

| **Table 2. Behavioural results: within-Subjects Effects and group interaction effects** | | | | |
| --- | --- | --- | --- | --- |
| **Effect** | **df** | **F** | ***p*** | **η²p (Effect Size)** |
| Type image (Neutral vs. Emotional) | 1, 74 | 5.46 | .022 | .067 |
| Type image × group | 2,74 | 1.48 | .233 | .038 |
| Time (Immediate vs. Delayed) | 1, 74 | 124.09 | <.001 | .620 |
| Time × group | 2,74 | .94 | .39 | .024 |
| Type image × Time | 1, 74 | 1.93 | .169 | .025 |
| Type image × time × group | 2,74 | 1.12 | .33 | .025 |
| Note. The table shows the mixed effects ANOVA SPSS results of the behavioural memory results (hits minus false alarms of emotional images vs neutral images in time point one or immediate vs time point two or delayed). df = degrees of freedom, F = F-statistic (from SPSS output), *p* = p-value (significance level), η²p (Partial Eta Squared) = Effect size (SPSS reports this in "Tests of Within-Subjects Effects"). | | | | |

| **Functional activations contrasts of interest. Uncorrected p values, threshold p<.005, cluster p(FDR) corrected. When applying small volume corrections, threshold p <.01 p(FEW-corrected)** | | | | | | |
| --- | --- | --- | --- | --- | --- | --- |
| **Table 3. Sanity check response larger than fixation cross (motor)** | | | | | |  |
| **Motor whole group study template (cl 491)** | | | |  |  |  |
| **Region** | **p(FWE-corr)** | **p(FDR-corr)** | **equivk** | **x** | **y** | **z** |
| Parahippocampus | <.001 | <.001 | 36832 | 30 | -22 | -12 |
|  |  |  |  | 22 | -12 | -13 |
|  |  |  |  | 32 | -40 | -19 |
|  | <.001 | <.001 | 22979 | -30 | -19 | -12 |
|  |  |  |  | -1 | 25 | 25 |
|  |  |  |  | -29 | -33 | -15 |
| Precuneus | 0.169 | 0.007 | 610 | 20 | -38 | 7 |
|  |  |  |  | 25 | -40 | 15 |
| Precentral (motor) | 0.002 | <.001 | 1236 | 42 | 16 | 35 |
| Sup motor area | 0.001 | <.001 | 1420 | -8 | 5 | 57 |
|  |  |  |  | -5 | 13 | 52 |
|  | 0.404 | 0.016 | 491 | 9 | 12 | 61 |
|  |  |  |  | 8 | 16 | 54 |
|  |  |  |  | -9 | -3 | 63 |
| Pineal body | 0.256 | 0.01 | 555 | 4 | -8 | -3 |
|  |  |  |  | -1 | 1 | 2 |
|  | 0.015 | 0.001 | 932 | 33 | 20 | -23 |
|  |  |  |  | 38 | 17 | -17 |
| Precentral | 0.016 | 0.001 | 927 | -42 | 14 | 35 |
|  |  |  |  | -34 | 21 | 35 |
|  | 0.004 | <.001 | 1143 | -37 | -6 | 49 |
|  |  |  |  | -36 | 1 | 44 |
|  | 0.142 | 0.006 | 633 | 42 | -1 | 48 |
|  |  |  |  | 36 | 5 | 46 |
|  |  |  |  |  |  |  |
| **Motor younger adults study template (cl 332)** | | | |  |  |  |
| **Region** | **p(FWE-corr)** | **p(FDR-corr)** | **equivk** | **x** | **y** | **z** |
| Parahippocampus | <.001 | <.001 | 28773 | 29 | -24 | -13 |
|  |  | <.001 |  | 32 | -40 | -19 |
|  |  | <.001 |  | 27 | -47 | -21 |
|  | <.001 | <.001 | 20056 | -29 | -31 | -17 |
|  |  | <.001 |  | -27 | -47 | -19 |
|  |  | <.001 |  | -31 | -19 | -12 |
| Precentral | <.001 | <.001 | 1534 | 43 | 20 | 31 |
|  | <.001 | <.001 | 1567 | 43 | 1 | 47 |
|  |  |  |  | 35 | 5 | 46 |
|  |  |  |  | 36 | -7 | 49 |
|  | 0.001 | <.001 | 1198 | -40 | 13 | 36 |
|  |  |  |  | -51 | 12 | 33 |
|  |  |  |  | -37 | 21 | 35 |
|  | <.001 | <.001 | 1855 | -42 | -5 | 47 |
|  |  |  |  | -34 | -10 | 49 |
|  |  |  |  | -32 | 2 | 54 |
| Superior motor area | <.001 | <.001 | 1690 | -6 | 18 | 52 |
|  |  |  |  | -7 | 5 | 58 |
|  |  |  |  | -12 | -1 | 61 |
| Superior motor | 0.251 | 0.008 | 502 | 9 | 16 | 54 |
| Frontal mid | 0.019 | 0.001 | 812 | 40 | 37 | 28 |
|  | 0.354 | 0.011 | 459 | -40 | 36 | 31 |
|  |  |  |  | -37 | 39 | 24 |
|  |  |  |  | -37 | 26 | 31 |
| Fusiform / lingual | 0.647 | 0.023 | 373 | 35 | 3 | -18 |
|  | 0.374 | 0.011 | 452 | 35 | 20 | -21 |
|  |  |  |  | 28 | 27 | -22 |
|  |  |  |  | 38 | 11 | -14 |
| Parietal sup | 0.689 | 0.024 | 362 | -22 | -49 | 38 |
|  |  |  |  | -25 | -42 | 32 |
|  |  |  |  |  |  |  |
| **Motor younger adults group template (cl 300)** | | | |  |  |  |
| **Region** | **p(FWE-corr)** | **p(FDR-corr)** | **equivk** | **x** | **y** | **z** |
| Lingual V1 | <0.001 | <0.001 | 30485 | 29 | -25 | -7 |
|  |  |  |  | 29 | -36 | -8 |
|  |  |  |  | 32 | -54 | -3 |
| Lingual | <0.001 | <0.001 | 20890 | -29 | -37 | -10 |
|  |  |  |  | -29 | -27 | -7 |
|  |  |  |  | -27 | -49 | -11 |
| Precentral | <0.001 | <0.001 | 1599 | 41 | 18 | 33 |
|  | <0.001 | <0.001 | 1819 | 42 | 5 | 50 |
|  |  |  |  | 29 | -12 | 53 |
|  | <0.001 | <0.001 | 2116 | -37 | 5 | 49 |
|  |  |  |  | -41 | -3 | 52 |
|  |  |  |  | -34 | -9 | 54 |
| Precenral mid | <0.001 | <0.001 | 1386 | -41 | 14 | 38 |
|  |  |  |  | -52 | 13 | 34 |
|  |  |  |  |  |  |  |
| Supplem motor | 0.149 | 0.005 | 556 | 9 | 17 | 57 |
|  | <0.001 | <0.001 | 1687 | -9 | 17 | 56 |
|  |  |  |  | -7 | 5 | 62 |
|  |  |  |  |  |  |  |
| Frontal mid | 0.005 | <0.001 | 967 | 40 | 38 | 29 |
|  |  |  |  | 39 | 32 | 36 |
|  | 0.094 | 0.003 | 609 | -40 | 36 | 30 |
|  |  |  |  | -38 | 26 | 32 |
| Fusiform | 0.178 | 0.005 | 535 | 36 | 15 | -17 |
|  |  |  |  | 41 | 7 | -11 |
| **Motor older adults study template (cl 459)** | | | |  |  |  |
| **Region** | **p(FWE-corr)** | **p(FDR-corr)** | **equivk** | **x** | **y** | **z** |
| Fusiform | <.001 | <.001 | 1610 | -29 | -26 | -12 |
|  |  |  |  | -30 | -17 | -12 |
|  |  |  |  | -21 | -13 | -12 |
|  |  |  |  | 23 | -20 | -12 |
| Parahippocampus | <.001 | <.001 | 10855 | 24 | -11 | -13 |
| **Motor older adults group template (cl 477)** | | | |  |  |  |
| **Region** | **p(FWE-corr)** | **p(FDR-corr)** | **equivk** | **x** | **y** | **z** |
| Fusiform | <0.001 | <0.001 | 1566 | -29 | -27 | -14 |
|  |  |  |  | -21 | -14 | -14 |
|  |  |  |  | -30 | -18 | -14 |
| Parhippocampus | <0.001 | <0.001 | 11188 | 24 | -12 | -15 |
|  |  |  |  | 31 | -22 | -14 |
| Fusiform |  |  |  | 31 | -58 | -11 |
| **Motor MCI study template (cl 474)** | | |  |  |  |  |
| **Region** | **p(FWE-corr)** | **p(FDR-corr)** | **equivk** | **x** | **y** | **z** |
| Parahippocampus | <.001 | <.001 | 2418 | 25 | -19 | -10 |
|  |  |  |  | 24 | -4 | -17 |
|  |  |  |  | 21 | -13 | -15 |
|  | 0.336 | 0.024 | 474 | -23 | -16 | -14 |
|  |  |  |  | -30 | -20 | -13 |
| Occipital Inferior | 0.078 | 0.007 | 651 | 45 | -50 | -7 |
|  |  |  |  | 48 | -59 | -7 |
|  |  |  |  | 36 | -61 | -4 |
| **Motor MCI group template (cl 525)** | | |  |  |  |  |
| **Region** | **p(FWE-corr)** | **p(FDR-corr)** | **equivk** | **x** | **y** | **z** |
| Parahippocampus | <0.001 | <0.001 | 2315 | 24 | -18 | -14 |
|  |  |  |  | 24 | -3 | -18 |
|  |  |  |  | 21 | -11 | -18 |
|  | 0.189 | 0.014 | 550 | -31 | -20 | -16 |
|  |  |  |  | -23 | -15 | -17 |
| Occipital inferior | 0.232 | 0.014 | 525 | 44 | -56 | -11 |
|  |  |  |  | 35 | -60 | -8 |
|  |  |  |  |  |  |  |
| **Table 4. Significant activations of the contrast Emotional Processing (Negative vs. Neutral scenes)** | | | | | | |
| **Emotional whole group study template (cl 534)** | | | |  |  |  |
| **Region** | **p(FWE-corr)** | **p(FDR-corr)** | **equivk** | **x** | **y** | **z** |
| Frontal lnf mid | <.001 | <.001 | 2891 | 54 | 32 | 35 |
|  |  |  |  | 46 | 50 | 11 |
|  |  |  |  | 52 | 50 | 25 |
|  |  |  |  | -13 | -12 | -2 |
|  |  |  |  | -13 | -1 | -4 |
| Frontal mid orb | <.001 | <.001 | 2728 | 1 | 66 | 3 |
|  |  |  |  | -4 | 72 | 11 |
|  |  |  |  | 2 | 57 | 0 |
| Frontal Sup medial | <.001 | <.001 | 1611 | 9 | 39 | 60 |
|  |  |  |  | 18 | 52 | 57 |
|  |  |  |  | 6 | 55 | 58 |
|  | <.001 | <.001 | 2296 | -8 | 65 | 52 |
|  |  |  |  | -4 | 50 | 59 |
|  |  |  |  | -6 | 58 | 47 |
| Frontal Inf Orb | <.001 | <.001 | 8695 | -44 | 44 | 5 |
|  |  |  |  | -53 | 43 | 26 |
|  |  |  |  | -52 | 37 | 20 |
| Frontal Inf Tri | 0.068 | 0.012 | 703 | 39 | 14 | 35 |
|  |  |  |  | 33 | 8 | 40 |
|  |  |  |  | 42 | 19 | 42 |
| Frontal Mid | 0.186 | 0.031 | 576 | -32 | 30 | 56 |
|  |  |  |  | -42 | 28 | 52 |
| Temporal Inf Lobe | <.001 | <.001 | 91628 | -44 | -35 | -26 |
|  |  |  |  | 58 | 18 | -3 |
|  |  |  |  | -44 | 42 | -3 |
| Lingual | <.001 | <.001 | 1757 | -16 | -36 | -7 |
|  |  |  |  | -11 | -25 | -10 |
|  |  |  |  | -24 | -32 | -8 |
| Cingulum Mid | 0.001 | <.001 | 1336 | -8 | -38 | 25 |
|  |  |  |  | -2 | -39 | 34 |
|  |  |  |  | -14 | -33 | 27 |
| Amygdala | 0.005 | 0.012 | 1055 | -28 | 12 | -4 |
|  |  |  |  | -25 | 25 | 9 |
|  |  |  |  | -26 | 24 | -1 |
| Caudate | 0.037 | 0.012 | 782 | -10 | 28 | 19 |
|  |  |  |  | -22 | 18 | 20 |
|  |  |  |  | -17 | 36 | 20 |
| Cerebellum | 0.707 | 0.092 | 384 | -34 | -28 | -56 |
| Occipital- Primary visual Cortex | <.001 | <.001 | 1446 | 13 | -54 | -11 |
| **Small volume corrections emotional whole group** | | | |  |  |  |
| Thalamus | 0.043 | 0.074 | 411 | -20 | -4 | 13 |
| **Emotional younger adults study template (cl 383)** | | | |  |  |  |
| **Region** | **p(FWE-corr)** | **p(FDR-corr)** | **equivk** | **x** | **y** | **z** |
| Frontal sup | <.001 | <.001 | 12065 | -11 | 55 | 60 |
|  |  |  |  | -5 | 75 | 28 |
|  |  |  |  | -5 | 71 | 44 |
| Lingual | <.001 | <.001 | 8445 | -13 | -34 | -7 |
|  |  |  |  | -6 | -44 | -1 |
|  |  |  |  | 14 | -54 | -12 |
| Occipital inferior | <.001 | <.001 | 6781 | 35 | -59 | -27 |
|  |  |  |  | 45 | -34 | -25 |
|  |  |  |  | 45 | -48 | -24 |
| Temporal superior | <.001 | <.001 | 1826 | 49 | 24 | -7 |
|  |  |  |  | 58 | 20 | -3 |
|  |  |  |  | 39 | 27 | -8 |
| Insula | <.001 | <.001 | 2294 | -24 | 25 | -1 |
|  |  |  |  | -29 | 10 | -7 |
|  |  |  |  | -32 | 21 | -14 |
| Cerebellum 9 | <.001 | <.001 | 1568 | -7 | -28 | -36 |
|  |  |  |  | 3 | -20 | -38 |
|  |  |  |  | 13 | -33 | -42 |
| Occipital inferior | <.001 | <.001 | 15913 | -36 | -61 | -30 |
|  |  |  |  | -32 | -66 | -24 |
|  |  |  |  | -14 | -45 | -31 |
| Temporal superior | <.001 | <.001 | 2471 | -60 | 2 | 1 |
|  |  |  |  | -54 | -1 | -8 |
|  |  |  |  | -47 | -4 | 1 |
| Frontal mid orb | <.001 | <.001 | 2487 | -28 | 37 | -19 |
|  |  |  |  | -44 | 42 | 4 |
|  |  |  |  | -45 | 42 | -4 |
| Temporal mid | <.001 | <.001 | 1932 | -53 | -27 | 8 |
|  |  |  |  | -64 | -34 | 1 |
|  |  |  |  | -45 | -22 | 2 |
| Occipital inferior | <.001 | <.001 | 5162 | -23 | -73 | 0 |
|  |  |  |  | -13 | -77 | 5 |
|  |  |  |  | -32 | -70 | 11 |
| Temporal mid | <.001 | <.001 | 2443 | 57 | -5 | -3 |
|  |  |  |  | 50 | -11 | -1 |
|  |  |  |  | 49 | -21 | 0 |
| Insula | 0.512 | 0.03 | 433 | 22 | 24 | -4 |
|  |  |  |  | 33 | 20 | -2 |
| Caudate | 0.002 | <.001 | 1224 | 12 | 31 | 19 |
|  |  |  |  | 27 | 12 | 18 |
|  |  |  |  | 22 | 21 | 17 |
| Hippocampus | 0.019 | 0.001 | 867 | 28 | 11 | -8 |
|  |  |  |  | 20 | 8 | -11 |
| Frontal Inf orb | 0.688 | 0.048 | 383 | 28 | 28 | -15 |
| Temporal pole inf | |  |  | 31 | 38 | -24 |
|  | |  |  | 25 | 31 | -23 |
| Caudate | 0.237 | 0.013 | 540 | -9 | 33 | 16 |
|  |  |  |  | -20 | 33 | 20 |
| Cerebellum 9 | <.001 | <.001 | 3895 | 11 | -47 | -45 |
| Cerebellum Crus | |  |  | 19 | -52 | -39 |
| Cerebellum 8 | |  |  | 29 | -30 | -50 |
| Frontal Inferior TrI | |  |  | -52 | 46 | 21 |
| Cingulum posterior | 0.382 | 0.021 | 476 | 1 | -39 | 35 |
| Precuneus |  |  |  | -6 | -44 | 39 |
| Temporal sup | 0.086 | 0.005 | 671 | 64 | -11 | 7 |
|  | |  |  | 66 | -18 | 13 |
|  | |  |  | 60 | -28 | 10 |
| Fusiform |  |  |  | 36 | -16 | -25 |
| Temporal sup | 0.015 | 0.001 | 901 | -52 | 24 | 1 |
| Temporal pole sup | 0.187 | 0.011 | 571 | 45 | 36 | -18 |
|  | |  |  | 42 | 29 | -21 |
| Frontal inf orb | 0.371 | 0.021 | 480 | 28 | 47 | -14 |
|  | |  |  | 30 | 42 | -5 |
|  | |  |  | 30 | 51 | -5 |
| Frontal mid orb | 0.092 | 0.005 | 661 | -22 | 25 | 47 |
|  |  |  |  | -27 | 23 | 55 |
| **Small volume corrections emotional younger adults study template** | | | | | |  |
| Amygdala | 0.004 | 0.018 | 572 | -27 | 11 | -6 |
| Hippocampus | 0.01 | 0.025 | 582 | 20 | 8 | -11 |
| **Emotional older adults study template (cl 417 )** | | | |  |  |  |
| **Region** | **p(FWE-corr)** | **p(FDR-corr)** | **equivk** | **x** | **y** | **z** |
| Frontal Inf Tri | 0.005 | 0.001 | 940 | -47 | 51 | 16 |
|  |  |  |  | -39 | 51 | 17 |
| Temporal Mid Lobe | 0.001 | <.001 | 1223 | -56 | -23 | -4 |
|  |  |  |  | -62 | -31 | -2 |
|  |  |  |  | -64 | -22 | -2 |
| Temporal Inf | 0.003 | 0.001 | 998 | -48 | -30 | -25 |
|  |  |  |  | -44 | -22 | -21 |
|  |  |  |  | -51 | -40 | -24 |
| Temporal Sup | <.001 | <.001 | 1873 | -56 | -30 | 14 |
|  |  |  |  | -48 | -33 | 17 |
|  |  |  |  | -58 | -40 | 9 |
| Precuneus | 0.348 | 0.035 | 448 | -10 | -38 | 22 |
| Occipital Inf | 0.015 | 0.002 | 811 | 37 | -66 | -15 |
|  |  |  |  | 41 | -59 | -22 |
|  |  |  |  | 32 | -70 | -8 |
|  | 0.086 | 0.022 | 610 | -40 | -65 | -10 |
|  |  |  |  | -41 | -63 | -18 |
|  |  |  |  | -36 | -69 | -1 |
| Occipital Mid | 0.096 | 0.022 | 598 | -50 | -49 | 24 |
|  |  |  |  | -42 | -58 | 22 |
|  |  |  |  | -46 | -48 | 17 |
| Cerebellum | <.001 | <.001 | 5522 | 20 | -45 | -49 |
|  |  |  |  | -20 | -43 | -45 |
|  |  |  |  | 19 | -40 | -41 |
| **Small Volume Corrections emotional older adults study template** | | | | | |  |
| No small structures significant (No SVC) | | | |  |  |  |
| **Emotional MCI study template (cl 820)** | | |  |  |  |  |
| **Region** | **p(FWE-corr)** | **p(FDR-corr)** | **equivk** | **x** | **y** | **z** |
| Temporal Mid | 0.017 | 0.01 | 844 | -56 | -42 | 5 |
|  |  |  |  | -53 | -51 | 2 |
| Occipital Inf | 0.021 | 0.01 | 820 | 49 | -42 | -20 |
|  |  |  |  | 43 | -45 | -25 |
|  |  |  |  | 54 | -38 | -12 |
| **Small volume corrections emotional MCI study template** | | | | |  |  |
| No small structures significant (No SVC) | | | |  |  |  |
| **Emotional: additional results** | | |  |  |  |  |
| **Emotional younger adults > older adults study template (cl 745)** | | | | |  |  |
| No suprathreshold clusters | | |  |  |  |  |
| **Emotional older adults > younger adults study template** | | | | |  |  |
| FDRC Inf. |  |  |  |  |  |  |
| No small structures significant (No SVC) | | | |  |  |  |
| **Emotional older adults > MCI study template** | | | |  |  |  |
| FDRC Inf. |  |  |  |  |  |  |
| No small structures significant (No SVC) | | | |  |  |  |
| **Emotional MCI > older adults study template** | | | |  |  |  |
| FDRC Inf. |  |  |  |  |  |  |
| No small structures significant (No SVC) | | | |  |  |  |
|  |  |  |  |  |  |  |
| **Table 5. Significant activations Remembered Images Larger than not Remembered Images (memory)** | | | | | | |
| **Memory whole group study template** | | |  |  |  |  |
| FDRC inf |  |  |  |  |  |  |
| **Small volume corrections memory whole group** | | | |  |  |  |
| Putamen | 0.005 | 0.008 | 665 | 19 | 29 | 6 |
| Parahippocampus | 0.121 | 0.136 | 206 | -32 | 1 | -17 |
|  | 0.102 | 0.136 | 225 | 26 | -16 | -12 |
|  |  |  |  |  |  |  |
| **Memory younger adults study template (cl 482)** | | | |  |  |  |
| **Region** | **p(FWE-corr)** | **p(FDR-corr)** | **equivk** | **x** | **y** | **z** |
| Precentral | <.001 | <.001 | 2416 | 35 | -6 | 41 |
|  |  |  |  | 39 | -6 | 51 |
|  |  |  |  | 46 | -6 | 42 |
| Parahippocampus | 0.165 | 0.018 | 558 | -33 | 0 | -15 |
|  |  |  |  | -36 | -11 | -10 |
| Parahippocampus | <.001 | <.001 | 2123 | 36 | -13 | -9 |
|  |  |  |  | 25 | -22 | -10 |
|  |  |  |  | 31 | -11 | -16 |
| Lingual | <.001 | <.001 | 2338 | -29 | -25 | -16 |
|  |  |  |  | -10 | -25 | -23 |
|  |  |  |  | -25 | -31 | -20 |
| Cuneus | 0.048 | 0.007 | 708 | 17 | -54 | 34 |
|  |  |  |  | 47 | -58 | 4 |
|  |  |  |  | 33 | -62 | -5 |
| Occipital mid | <.001 | <.001 | 1776 | -21 | -72 | -5 |
|  |  |  |  | -35 | -70 | -4 |
|  |  |  |  | -17 | -68 | -13 |
| Occipital mid R | 0.306 | 0.033 | 482 | 38 | -61 | 2 |
|  |  |  |  | 47 | -58 | 4 |
|  |  |  |  | 33 | -62 | -5 |
| **Small volume corrections memory younger adults study template** | | | | | |  |
| Putamen | 0.111 | 0.151 | 272 | 18 | 25 | 5 |
| **Memory older adults study template (cl 627)** | | | |  |  |  |
| **Region** | **p(FWE-corr)** | **p(FDR-corr)** | **equivk** | **x** | **y** | **z** |
| Putamen | 0.058 | 0.027 | 627 | 24 | 22 | 3 |
| **Small volume corrections memory older adults** | | | |  |  |  |
| LC | 0.082 | 0.936 | 4 | -6 | -8 | -27 |
| Putamen | 0.047 | 0.041 | 335 | -23 | 32 | 8 |
|  | 0.002 | 0.004 | 725 | 24 | 22 | 3 |
| **Memory MCI study template** | | |  |  |  |  |
| Inf |  |  |  |  |  |  |
| No SVC |  |  |  |  |  |  |
| **Memory: additional results** | | |  |  |  |  |
| **Memory older adults > MCI study template** | | | |  |  |  |
| Inf |  |  |  |  |  |  |
| **Small volume corrections memory older adults > MCI study template** | | | | | |  |
| LC | 0.081 | 0.936 | 4 | -6 | -8 | -27 |
| **MCI > Older adults** | |  |  |  |  |  |
| Inf |  |  |  |  |  |  |
| No SVC |  |  |  |  |  |  |
| **Memory younger adults > older adults study template (cl 637)** | | | | |  |  |
| **Region** | **p(FWE-corr)** | **p(FDR-corr)** | **equivk** | **x** | **y** | **z** |
| Cerebellum 7b | <.001 | 0.01 | 1360 | -44 | -14 | -44 |
|  |  |  |  | -43 | -28 | -54 |
|  |  |  |  | -38 | -17 | -57 |
| Precuneus | 0.002 | 0.01 | 1130 | 11 | -58 | 34 |
|  |  |  |  | 20 | -52 | 39 |
|  |  |  |  | 9 | -64 | 23 |
| Precentral | 0.096 | 0.015 | 644 | 35 | -1 | 54 |
|  |  |  |  | 37 | -5 | 45 |
|  |  |  |  | 43 | -4 | 53 |
| **Memory younger adults > MCI study template (cl 716)** | | | | |  |  |
| **Region** | **p(FWE-corr)** | **p(FDR-corr)** | **equivk** | **x** | **y** | **z** |
| Cuneus | 0.055 | 0.015 | 716 | 17 | -53 | 35 |
|  |  |  |  | 27 | -51 | 35 |
| Fusiform | 0.014 | 0.006 | 889 | -29 | -25 | -16 |
|  |  |  |  | -21 | -25 | -13 |
|  |  |  |  | -31 | -19 | -11 |
| Precentral | 0.002 | 0.002 | 1156 | 37 | -5 | 47 |
|  |  |  |  | 46 | -2 | 55 |
|  |  |  |  | 34 | -11 | 39 |
| **Small volume corrections memory younger adults > older adults study template** | | | | | | |
| No SVC |  |  |  |  |  |  |
| **Memory older adults > younger adults study template** | | | | |  |  |
| Inf |  |  |  |  |  |  |
| .01 Inf - no SVC | |  |  |  |  |  |
| **Memory MCI > younger adults study template** | | | |  |  |  |
| Inf |  |  |  |  |  |  |
|  |  |  |  |  |  |  |

References supplementary:

1.	Watanabe T. Neuromelanin? MRI of Noradrenergic and Dopaminergic Neurons. *Magnetic Resonance Imaging*. 2023;58(1):328-329. doi:10.1002/jmri.28479

2.	Priovoulos N, Jacobs HIL, Ivanov D, Uludağ K, Verhey FRJ, Poser BA. High-resolution in vivo imaging of human locus coeruleus by magnetization transfer MRI at 3T and 7T. *NeuroImage*. 2018;168:427-436. doi:10.1016/j.neuroimage.2017.07.045

3.	Fernandes P, Regala J, Correia F, Gonçalves-Ferreira AJ. The human locus coeruleus 3-D stereotactic anatomy. *Surg Radiol Anat*. 2012;34(10):879-885. doi:10.1007/s00276-012-0979-y

4.	German D, Walker B, Manaye K, Smith W, Woodward D, North A. The human locus coeruleus: computer reconstruction of cellular distribution. *The Journal of Neuroscience*. 1988;8(5):1776-1788. doi:10.1523/JNEUROSCI.08-05-01776.1988

5.	Keren NI, Taheri S, Vazey EM, et al. Histologic validation of locus coeruleus MRI contrast in post-mortem tissue. *NeuroImage*. 2015;113:235-245. doi:10.1016/j.neuroimage.2015.03.020

6.	German DC, Walker BS, Manaye K, Smith WK, Woodward DJ, North AJ. The human locus coeruleus: computer reconstruction of cellular distribution. *J Neurosci*. 1988;8(5):1776-1788. doi:10.1523/JNEUROSCI.08-05-01776.1988

7.	Ritchey M, Dolcos F, Cabeza R. Role of Amygdala Connectivity in the Persistence of Emotional Memories Over Time: An Event-Related fMRI Investigation. *Cerebral Cortex*. 2008;18(11):2494-2504. doi:10.1093/cercor/bhm262

8.	Zhunussova A, Loane C, Kurt E, et al. Changes in Task‐evoked Pupil Dilation during Emotional Memory as a Marker of Noradrenergic Function in Healthy Aging and MCI. *Alzheimer’s &amp; Dementia*. 2025;21(S7):e108410. doi:10.1002/alz70861_108410

1. Throughout the Supplementary and manuscript we use the terms Locus Coeruleous (LC) contrast and LC intensity indistinctively. These are a proxy for LC structural health and possibly an indirect mesure of LC integrity (see discussion on the topic in Reference ^1^ of this Supplementary). The term LC integrity is used only to reference studies that used histology or to make hypothesis based on our LC structural findings.
